# Recent advances in defect engineering vacancies of indium oxide nanomaterials for CO_2_ photoreduction

**DOI:** 10.1039/d6ra05293b

**Published:** 2026-07-28

**Authors:** Julien Laurent, Viet Van Pham

**Affiliations:** a Advanced Materials and Applications Research Group, HUTECH University Ho Chi Minh City 70000 Vietnam pv.viet@hutech.edu.vn; b CIRTech Institute, HUTECH University Ho Chi Minh City 70000 Vietnam

## Abstract

Oxygen vacancies have transformed In_2_O_3_ from a conventional semiconductor into one of the most promising photocatalysts for solar-driven CO_2_ reduction. Rather than acting as isolated defects, they define the electronic structure, catalytic active sites, and reaction pathways that govern photocatalytic performance. This review highlights the emerging transition from increasing vacancy concentration to engineering vacancy architecture through precise control of defect location, distribution, clustering, and stability. We critically discuss recent advances in defect engineering, characterization techniques, and theoretical understanding while identifying current limitations in defect quantification and catalyst evaluation. Finally, key challenges, including *operando* characterization, quantitative defect descriptors, long-term stability, selective C_2+_ production, and scalable catalyst design, are presented. By integrating these perspectives, this review provides a framework for the rational engineering of vacancy-controlled In_2_O_3_ photocatalysts for efficient and durable CO_2_ conversion.

## Abstract 2

This review discusses about the role of OVs of In_2_O_3_ in enhancing the photocatalytic efficiency of CO_2_ reduction in fuels, and highlights methods/strategies for creating defects in In_2_O_3_ aimed at improving its catalytic activity.

## Graphical abstract

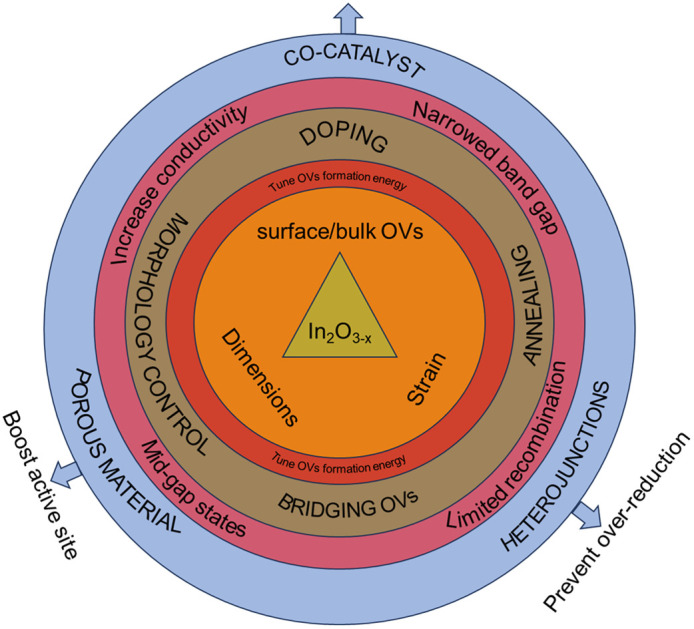

## 1. Introduction

The continuous rise in atmospheric carbon dioxide (CO_2_) concentration driven primarily by anthropogenic activities such as fossil fuel combustion, industrial production, and land-use change has become one of the most pressing environmental challenges of our era. Over the past three decades, atmospheric CO_2_ levels have increased from approximately 350 ppm to more than 420 ppm, correlating with a global temperature rise of nearly 1 °C and accelerating climate-related phenomena such as sea-level rise, melting of polar ice, and desertification. Despite international agreements such as the Kyoto Protocol and the Paris Agreement designed to limit greenhouse gas emissions, current global efforts remain insufficient to stabilize CO_2_ concentrations and achieve carbon neutrality. In this context, strategies focusing on both carbon emission reduction and resource circularity have gained increasing attention. Among emerging technologies, CO_2_ photoreduction the solar-driven conversion of CO_2_ into value-added fuels and chemicals offers a sustainable route to close the anthropogenic carbon cycle. By integrating renewable energy utilization with greenhouse gas mitigation, this process represents a promising pathway toward a low-carbon and climate-resilient future.^1,2^

While CO_2_ photoreduction offers an appealing route to carbon neutrality, the process remains fundamentally constrained by the exceptional thermodynamic stability of the CO_2_ molecule (Δ*G*^f^_0_ = −394.4 kJ mol^−1^).^3^ The fundamental challenge is the high activation energy (approx. 750 kJ mol^−1^) required to dissociate the robust C=O bonds, which necessitates a substantial energy input and yields sluggish CO_2_RR kinetics.^4–6^ Further technical hurdles include: the complex multi-electron and transfer pathways of the CO_2_RR,^7^ resulting in poor selectivity due to the formation of a product mixture (CO, CH_4_, CH_3_OH, *etc.*) and competition from unwanted side reactions and limitations in designing highly efficient and stable catalysts capable of facilitating the multiple C–C coupling steps required for producing desirable long-chain hydrocarbons.^4,6,8^ Mohata *et al.* acknowledge that CO_2_RR consistently competes with the HER. In addition, they noted that while HER can be significantly suppressed, it cannot be entirely eliminated.^9^ To overcome CO_2_'s thermodynamic and kinetic limitations, photocatalysis has emerged as an especially attractive strategy, because it harnesses renewable solar energy by mimicking the natural photosynthesis.^10,11^ Unlike electrocatalysis or thermo-catalysis, the photocatalysis operates under mild conditions (avoiding high temperature, pressure, or intense electrical input) and requires no external energy input. This leads to this approach becoming an optimal, sustainable, and clean solution for mitigating excessive emissions, energy crises, and global warming.^12^

**Fig. 1:**
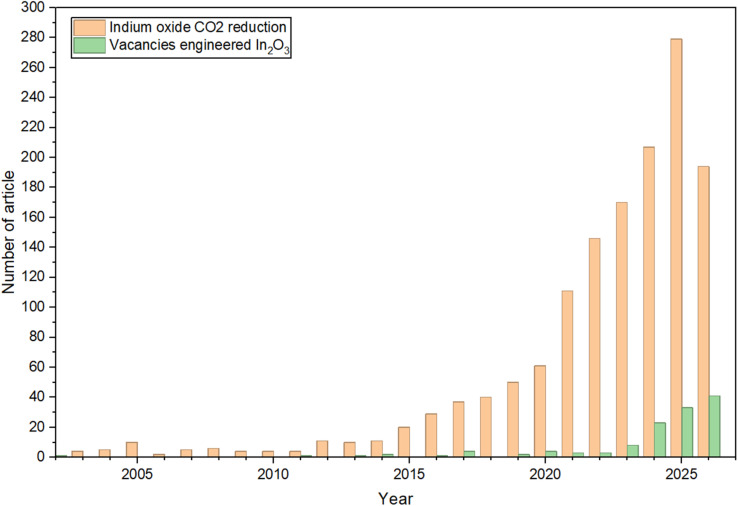
Statistic of research articles from ScienceDirect data May 2026. To analyze the publication trends from 2000 to May 2026, a systematic literature search was conducted on ScienceDirect. The annual volume of published articles was determined by refining the search results year-by-year using the database's filter checkboxes. This data collection focused on two distinct search queries: “indium oxide CO_2_ reduction” and “vacancies engineered In_2_O_3_”. The resulting annual article counts were then compiled to generate a chronological graph illustrating the research growth over time for each topic.

Since 2015, studies on indium oxide (In_2_O_3_) for CO_2_RR has received considerable attention, as evidenced by the continuously increasing number of publications on this material (Fig. 1). Indeed, In_2_O_3_ is a standout wide-gap based photocatalyst^13^ for converting CO_2_ to useful products like methanol using visible light region,^14^ thanks to its exceptional electronic and structural features. Furthermore, the material's conduction and valence bands are perfectly aligned with the energy requirements for water oxidation and CO_2_RR, providing an ideal platform for artificial photosynthesis.^15^ Critically, In_2_O_3_'s inherent tendency to form OVs allows researchers to employ defect engineering a method used to tune the material's electronic structure and raise the *E*_f_ which dramatically boosts its reduction capability and leads to superior selectivity toward methanol formation.^8,15^ Expanding on oxide catalysts, Wu *et al.*, synthesize a bimetal oxysulfide catalyst. By modulating of hetero-valent metal states they were able to precisely control the concentration of intrinsic OVs. This enrichment of OVs significantly boosted photocatalytic activity and stability by enhancing both charge separation and carrier mobility.^16^ Building upon these intrinsic properties, recent studies have focused on engineering OVs within In_2_O_3_ to further enhance its photocatalytic performance, selectivity, and stability in CO_2_RR. This engineering is critical because OVs strongly modulates the material's electronic structure and may significantly increases active sites.^17,18^

OVs significantly influence the electronic and structural properties of In_2_O_3_, which is critical for enhancing its photocatalytic performance, especially under visible light irradiation. Electronically, OVs narrow the bandgap, which broadens light absorption and improves the visible light photocatalysis. They also introduce defect energy levels (mainly In 6p states) within the bandgap that create a two-step electron transition pathway (valence band → defect level → conduction band). This mechanism drastically lowers the energy needed for visible light excitation. Furthermore, OVs function as electron traps, effectively capturing photoinduced electrons and thereby improving charge carrier separation by reducing the recombination of photogenerated electrons and holes. As demonstrated by Buy *et al.* (2021) in an SnO_2−*x*_/g-C_3_N_4_ composite, the presence of OVs within SnO_2−*x*_ effectively suppresses electron–hole pair recombination.^19^ However, Ye *et al.* (2019) cautioned that an overabundance of these defects can trigger the opposite effect.^11^

Structurally and chemically, it has been demonstrated that in some cases, OVs act as electron-rich active sites that boost the material's affinity for reactant molecules, enhancing its chemical adsorption and overall catalytic activity. For instance, Thang *et al.* (2021) developed an Ag-gC_3_N_4_/ZnO 1D nanorod heterojunction for CO_2_ photoreduction. They demonstrated that OVs compensate for the wide band gap of ZnO. Furthermore, in such configuration with silver atom, the presence of OVs leads to enhanced light sensitivity and superior carrier separation. The source also emphasized that ZnO-OVs electrostatically interacts with acidic CO_2_ molecule to form surface bound in CO_2_RR.^12^ Conversely, Thang *et al.* demonstrated that excessive quantities of OVs actually suppress photocatalytic activity.^20^ Another characteristics of OVs are their formation induces lattice distortions and strain (tensile or compressive) on surrounding atoms, which can create more reactive surface regions and positively impact catalytic kinetics.^17,18^

**Fig. 2:**
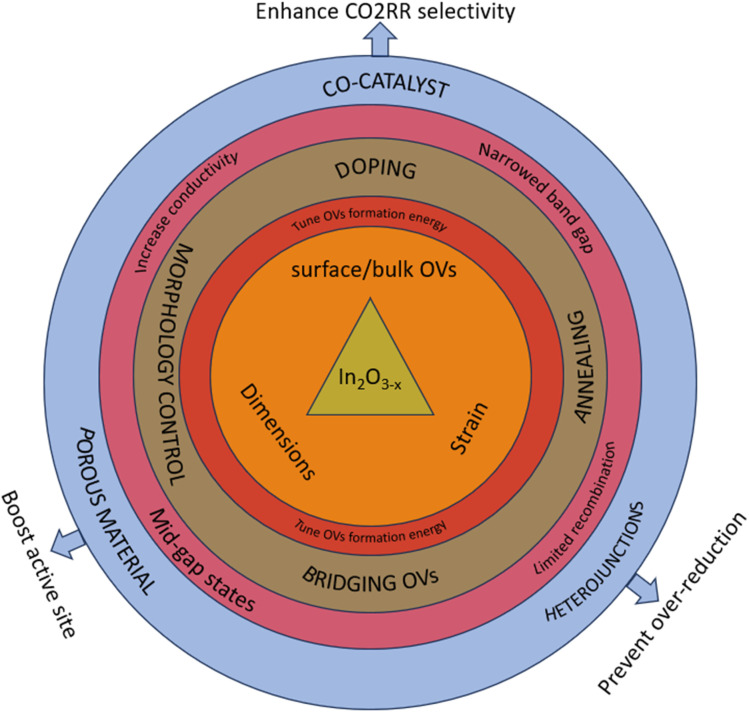
Visual map of the article's content.

Despite its high capacity, In_2_O_3_ is an underexplored catalyst, particularly regarding the engineering of its OVs, which are promising sites for CO_2_RR. The current research landscape suffers from several critical gaps: the intrinsic function and atomic-level mechanisms of OVs remain elusive and controversial. As previously noted with related catalytic systems, the impact of OVs remains highly contingent upon the material's structural environment. Providing a comprehensive perspective on these underlying mechanisms is essential for elucidating true OV functionality. Also, researchers lack the fine control necessary to optimize OVs concentration and location (*e.g.*, differentiating between bulk and surface OVs or designing bridging OVs). Moreover, there is no fundamental understanding of how structural factors (lattice strain, size, and dimension) impact OVs stability. Addressing these fundamental gaps especially achieving better stability and control is essential not only to unlock In_2_O_3_'s full potential but also to overcome the significant challenge of producing high-value C_2+_ chemicals. The present review is divided in five sections and aims to provide a comprehensive understanding of how OVs govern the photocatalytic reduction of CO_2_ over In_2_O_3_ and its modified derivatives (Fig. 2): (i) introduction of vacancies defect in indium oxide and their properties, (ii) the role of these vacancies for CO_2_ photoreduction focusing on 3 main approaches (tuning of the electronic structure, charge separation, and CO_2_ adsorption properties), (iii) overview of engineering strategies of OVs including thermal treatment, doping and novel methods, (iv) analytical methods of OVs identification, (v) synergistic strategies for high In_2_O_3_ catalyst performance over CO_2_ reduction (heterostructure design, structure–activity relationship linking defect chemistry to catalytic performance, role of metal and oxide co-catalysts in enhancing vacancy stability and reaction selectivity). Overall, we highlight emerging concepts such as strain engineering, SFLPs, and *in situ* characterization, providing insights into the future design of efficient, defect-controlled photocatalysts for sustainable CO_2_ conversion.

## 2. Overview of In_2_O_3_ and vacancies of In_2_O_3_

**Fig. 3:**
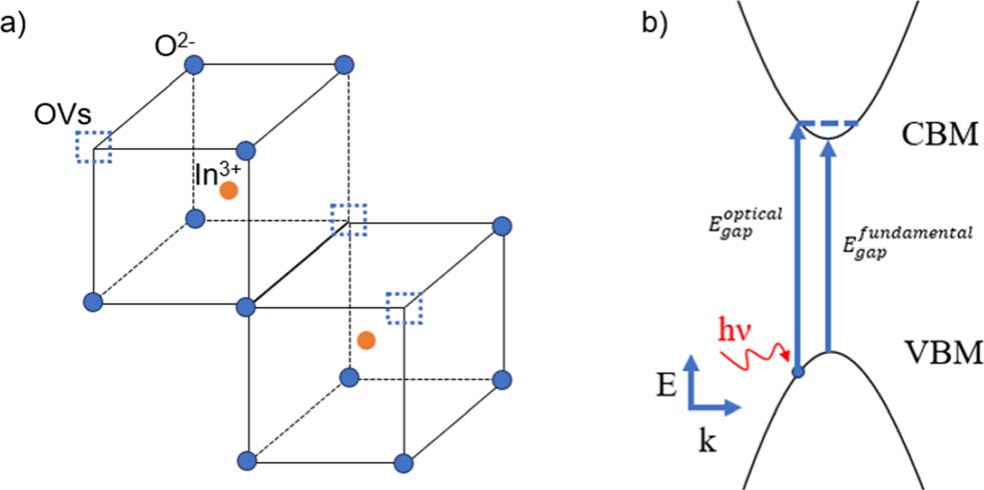
(a) Reduced indium oxide lattice. (b) Band gap structure illustration, adapted from ref. 25 with permission from IOP under the Creative Commons Attribution 3.0 License,^25^ copyright 2013.

In_2_O_3_ is a highly versatile material used as both an n-type semiconductor and a TCO.^21,22^ Its fundamental structure is the C-type rare-earth type, which closely resembles fluorite and allows for easy structural transformation.^22^ In_2_O_3_ exists in several crystal forms (polymorphs) determined by ambient conditions. The cubic (bixbyite) phase is the most common and stable form at low temperatures and ambient pressure, typically featuring a unit cell dimension of 10 angstroms and the stable In (222) crystalline plane (Fig. 3a).^22,23^ Other phases include the rhombohedral (corundum) form, which stabilizes under modest pressure, and a metastable orthorhombic phase observed at high temperatures and pressures.^24^

In_2_O_3_ is recognized for its high electrical conductivity at room temperature. This conductance is primarily due to oxygen vacant point defects (OVs), which are the dominant intrinsic defects.^22^ These OVs defects create shallow donor states located just below the conduction band (CB) minimum, placing the *E*_f_ correctly for an n-type material.^26^ In_2_O_3_ has a wide optical bandgap typically observed around 3.7 eV. However, the material shows a fundamental direct forbidden bandgap around 2.9 eV (cubic bixbyite) meaning the lowest-energy electronic transition is prohibited by symmetry^21,27,28^ (Fig. 3b). Its typical band structure and intrinsic defect properties make the indium oxide a good candidate for catalysis due to (i) intrinsic electron regulation;^29^ (ii) electron accumulation at the surface for all polymorphic phase;^27^ (iii) transparency regarding visible and near IR light; (iv) catalytic activity governed by redox reaction (PRO material) and controlled by defect sites.^22^

Combining a moderate bandgap and high carrier mobility with chemical robustness, In_2_O_3_ surpasses wide-gap UV-active oxide like TiO_2_,^30^ Nb_2_O_5_,^31^ and SnO_2_.^19^ It also offers distinct advantages over other materials, including greater stability than Bi_2_WO_6_,^11^ better photo corrosion behavior than ZnO,^32^ and superior CO_2_ activation compared to SrTiO_3_.^33^ While these intrinsic properties determine the baseline photocatalytic response, further enhancement largely depends on the deliberate control of native defects, particularly OVs, which modulate both the electronic structure and surface reactivity of In_2_O_3_. Meanwhile, the vacancies of In_2_O_3_ can also serve as active sites for catalysis, as they attract and interact with other molecules, improving the material's performance in chemical reactions. Overall, this vacancy tunes the optoelectronic structure and surface reactivity, making them central to CO_2_ photoreduction.

### 2.1. Oxygen vacancies

OVs are the typical point defects in In_2_O_3_ photocatalyst, which play a significant role in various catalytic reactions by influencing the electronic structure and activity of materials. They are considered active sites for the adsorption and activation of various molecules, including CO_2_, H_2_O, and CH_4_.^8,34^ These defects play a crucial and multifaceted role in enhancing the properties and performance of various materials especially In_2_O_3_ by electronic structure modulation, particularly in catalytic applications.^35–37^ Besides, OVs can act as donor levels, supplying free electrons to the conduction band, thereby optimizing the electron structure, electrical properties, and improving electron mobility.^37^Table 1 shows the main properties and characteristic of those OVs.

**Table 1:** Some OVs characteristics

OVs properties	Experimentation purpose	Advantages	Disadvantages	Ref.
Concentration of OVs	3In_2_O_3_-OVs samples, with different concentration	Moderate concentration: increase adsorption and activation for CO_2_, leading to suppress RWGS	CO_2_ adsorption effectiveness is not linear in concentration	38
Narrow the material's bandgap	Generating new energy level below CBM	Improve light absorption performance	None	37 and 39
Trapping site	Promoting effective separation and transfer of charge carriers	Avoiding recombination	Excessive OVs lead to overreduction and catalyst deactivation	40–42
Covalent bond	S-scheme WO_3_-OVs/In_2_S_3_	Chemical bonds between OVs act as electrons “bridge” that improves charge separation and photocatalytic activity	None	39
Local electronic regulation of metal active site	Affecting the d band	Vacancies lower the coordination of metal cations (In^3+^:InVO_4_ and Zn^2+^:ZnO) leading to suppressed side reaction	Need a precise tailoring: CO_2_ interaction may be weak or too strong	43

**Fig. 4:**
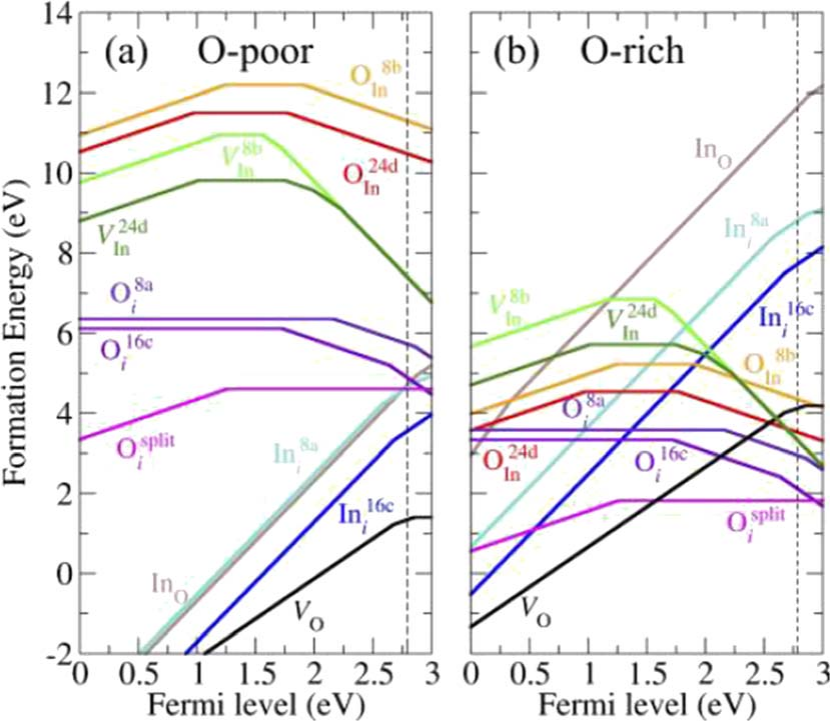
Formation energy as a function of *E*_f_ position for native point defect in indium oxide. The zero of *E*_f_ corresponds to the VBM and the dashed line indicates the CBM, reproduced from ref. 45 with permission from American Physical Society,^45^ Copyright 2019.

Moreover, OVs can exist in three main oxidation states OVs^2+^, OVs^+^ and OVs^0^ depending on the position of the *E*_f_. The oxidation state of each of these vacancies depends on In_2_O_3_ growth condition either oxygen poor (O-poor) or oxygen rich (O-rich) condition. According to the diagrams shown in Fig. 4, under O-poor conditions (left), the formation energy of OVs (black line) is very low and even becomes negative at low *E*_f_, meaning that OVs form easily and dominate the defect landscape. This naturally produces n-type behavior. In contrast, under O-rich conditions (right), the formation energy of OVs is much higher, so OVs are strongly suppressed; instead, indium vacancies and oxygen interstitials become more favorable. The slope changes in each line correspond to different charge states, so the position of the *E*_f_ dictates which charge state is stable.^44^ The Table 2 shown the properties of each oxidation states on indium oxide.

**Table 2:** Oxidation vacancies states in indium oxide

Oxidation states	Results	Ref.
OVs^0^	No conductivity contribution	44
	Stability condition	
	*E*_f_ > 0.06 eV above CBM	
OVs^+^	Widespread, moderate n-type	
	Stability condition	
	−0.11 eV < *E*_f_ < 0.06 eV (relative to CBM)	
OVs^2+^	Compensation center (*E*_f_ low) barrier to p-type doping	

#### 2.1.1. Surface and bulk oxygen vacancies

**Fig. 5:**
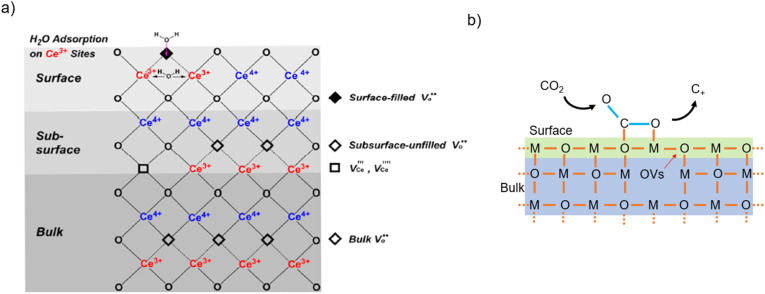
(a) Surface, sub-surface and bulk OVs, reproduced from ref. 46 with permission from American Chemical Society,^46^ copyright 2019. (b) Principle of OVs behavior near the surface for CO_2_ reduction.

The location of these OVs, whether on the surface or in the bulk of the material, plays a critical role in determining their specific effects. Surface OVs are typically located in the outermost atomic layers of a material and often have a lower formation energy compared to bulk vacancies (Fig. 5a).^46^ Bulk OVs, on the other hand, are situated deeper within the material's crystal lattice.^47,48^ OVs tend to migrate towards the surface, increasing surface OVs concentration, as surface OVs are generally more stable than bulk OVs. The migration of vacancies to the surface is due to energetic and thermodynamic concern. Indeed, surface often have lower coordination number and under coordinated metal atoms, which make then energetically favorable site for OVs compared to the bulk, in addition, the formation energy of OVs is usually lower at or near the surface, so vacancies created in the bulk tend to migrate outward (Fig. 5b).^35,49^

Surface OVs are highly advantageous due to their function as active sites for adsorption and catalysis.^37,47^ They provide a source of free electrons that not only improves conductivity but also lowers the energy barriers required for gas adsorption.^37^ Furthermore, these vacancies can significantly enhance photocatalysis by narrowing the material's bandgap and improving the separation of charges.^47^ They also contribute to stabilizing the surface structure and creating electron accumulation layers, which are essential for catalytic activity.^50^ However, a drawback is that an overabundance of surface OVs can act as charge recombination centers, which reduces photocatalytic efficiency and can also destabilize the material's surface, leading to poor stability under operational conditions.^47^ Also, over concentrated OVs can lead to catalyst deactivation. This happens through a process called agglomeration, where the vacancies clump together. Thus, it is important to tune surface OVs to get the best properties. For example, a study by Ye *et al.* (2023) on beta-Gallium Oxide (β-Ga_2_O_3_) showed that while a small number of OVs initially boosts catalytic activity, this effect is temporary. With time, the OVs agglomerate, creating an inactive, amorphous layer on the catalyst's surface that blocks active sites and reduces its effectiveness.^36^

In contrast to surface OVs, which are reasonably well-understood in the literature, bulk vacancies involve several controversial perspectives. On one hand, certain studies suggest that bulk OVs act as detrimental recombination centers, thereby lowering charge separation efficiency. On the other hand, alternative viewpoints emphasize their ability to induce additional photoexcitation, which enhances overall carrier density. Despite these conflicting roles, it is generally agreed that bulk OVs effectively alter the electronic band structure by narrowing the bandgap.^51,52^

Despite this, they are often considered harmful for charge transport because they can trap carriers and serve as recombination centers. Unlike surface OVs, bulk vacancies are more difficult to manage and remove, often requiring harsh treatments like annealing or plasma.^35^ For this reason, a balanced approach that combines the reactivity of surface OVs with the controlled electronic benefits of bulk OVs typically yields the best overall performance.^46,47^

Removing bulk OVs typically requires harsh conditions like high temperature annealing, but can be facilitated by hydrogen dopants that lower migration barriers and repel OVs.^35^ Beyond the spatial distribution of vacancies, their formation and stability are also highly sensitive to structural parameters such as size, dimension, and strain.

#### 2.1.2. Role of dimension and strain

Structural factors like dimension and strain are critical to the performance of MO_*x*_ catalysts because they influence the stability and formation of OVs, which are vital for catalytic activity. Dimension, particularly at the nanoscale, improves catalytic performance by increasing surface area, exposing more active sites, and enhancing OVs stability. At the same time, strain in the crystal lattice affects how easily OVs can form by modifying the local atomic and electronic structures. A comprehensive understanding of how a catalyst's size, strain, and surface structure impact OVs stability is essential for the rational design of more efficient and durable catalysts.^53^

**Fig. 6:**
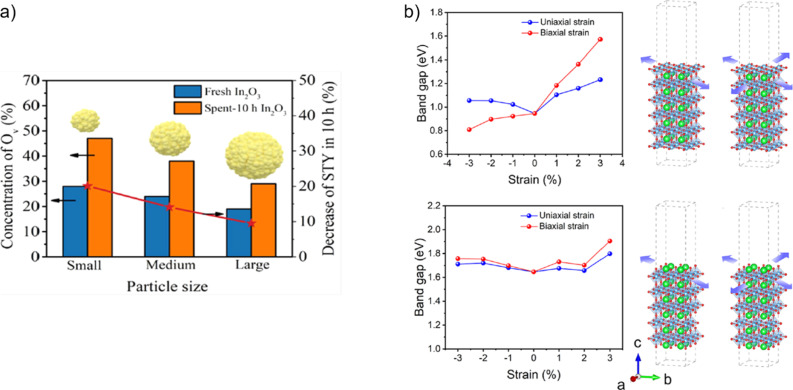
(a) Particle size effect on OVs concentration, reproduced from ref. 58 with permission from American Chemical Society,^58^ copyright 2023. (b) Effect of strain on bandgap and representation of uniaxial and biaxial strain TiO_2_-terminated STO slab, reproduced from ref. 59 with permission from Elsevier under the terms of the Creative Commons Attribution-Non Commercial-No Derivatives License,^59^ copyright 2024.

Numerous studies report an inverse relationship between nanoparticle size and OVs concentration (Fig. 6a). As particle size decreases, the increasing surface-to-volume ratio generates a higher density of surface defects specifically OVs which enhances the catalytic conversion of CO_2_ into CO or methanol. Conversely, larger nanoparticles tend to stabilize OVs, while this results in lower catalytic activity, it effectively prevents over-reduction. Consequently, optimizing catalytic performance requires balancing the trade-offs between particle size and OV density.^54,55^

The shape of In_2_O_3_ particles, known as their crystallographic structure, is a crucial factor in determining the number of surfaces OVs. Different crystal faces, or facets, have unique atomic arrangements that affect the ease with which these vacancies form. For example, Liu *et al.* (2015) found that the high-energy {110} facets on In_2_O_3_ are more likely to form OVs than the lower-energy {100} facets. This is because facets with higher surface energy (*γ*) require less energy to create a vacancy. The surface energy sequence for the body-centered cubic (bcc) structure of In_2_O_3_ is *γ*{111} < *γ*{100} < *γ*{110}, confirming that {110} facets possess the highest surface energy and therefore the highest concentration of OVs. While particle size affects the number of exposed OVs, lattice strain governs their energetic stability and migration, as discussed next.

Strain, a structural distortion within a material, can significantly manipulate the formation and distribution of OVs. The energy required to form OVs and their preferred locations are highly dependent on the type of strain applied (uniaxial *vs.* biaxial, tensile *vs.* compressive, static *vs.* fluxional) and the vacancy's position (surface, bulk). Studies demonstrate that OVs induce significant lattice distortion. Consequently, mechanical strain can be used to manipulate OVs by modulating their formation energy. For instance, while compressive strain increases the formation energy of surface OVs making them less thermodynamically stable uniaxial tensile strain lowers it. As a result, the generation of OVs is significantly more favorable under tensile strain conditions.^56,57^ This modification ultimately alters the electronic properties of the material as shown in Fig. 6b.

There are two distinct types of strain, including static strain and fluxional strain. Table 3 summarizes the effect of the environment to vacancy behavior and formation of the types of strain.

**Table 3:** OVs-induced distortions under different environments

Environment	Vacancy behavior	Static strain	Fluxional strain
Vacuum	Moderate concentration leading to stable surface	Strong with alternating tensile and compressive	Low concentration located mainly at step edge
Oxidizing	Continuous creation/annihilation leading to dynamic surface	Medium – surface localized	Very high
			Spread across the surface
Reducing	Very high concentration leading to surface unstable	Uniform concentration	High at step edge
			Cation migration

In short, OVs serve as a highly tunable lever for modulating the electronic structure and catalytic performance of In_2_O_3_ (Table 4). Successfully optimizing OVs requires a careful balance of surface-to-bulk defect ratios, particle size effects, and lattice strain to maximize reactivity while preventing over-reduction and catalyst deactivation. However, focusing exclusively on anion-site defects provides an incomplete picture of the crystal lattice. To build a holistic view of defect engineering, the next section shifts attention to the cation sub-lattice to evaluate the distinct catalytic environments introduced by indium vacancies.

**Table 4:** Structural factors influencing OVs formation and catalytic behavior in In_2_O_3_

Parameter	Effect on structure	Consequence on OVs	Impact on CO_2_ photoreduction
Dimension (particle size)	Smaller particles have higher surface/volume ratio	Increase surface OVs, but risk of over-reduction	High initial activity; stability trade-off
Facet orientation	{110} facets lead to undercoordinated In atoms	Drop OVs formation energy	Enhanced CO_2_ activation *via* bent adsorption geometry
Tensile strain	Bond stretching, lower orbital overlap	Drop OVs formation energy	Improved reducibility and activity
Compressive strain	Bond compression, higher overlap	Increase OVs formation energy	Stabilizes lattice, reduces reactivity
Fluxional strain field	Dynamic expansion/contraction at step edges	Enables OVs formation and migration in real time	Sustained reactivity and defect regeneration under light

### 2.2. Indium vacancies and cation deficiencies

Indium vacancies (VIn) are less common compared to OVs. VIn can exist in various charge states, including +1, neutral, −1, −2, and −3.^45^ Moreover, the removal of an indium atom to form a VIn results in six oxygen dangling bonds within the crystal lattice, leading to a total of three holes (missing electrons). These dangling bond states combine into partially occupied gap states with three holes.^45^ For instance, the V3–In state, where the VIn has a 3− charge, has a low formation energy under O-rich conditions and when the *E*_f_ is near the CBM. In this scenario, V3–In acts as a compensating center in n-type In_2_O_3_, particularly in TCO films with high carrier concentrations. This indicates that these vacancies participate in balancing the overall charge of the material. However, in moderately n-type doped In_2_O_3_, oxygen interstitials are the primary lowest energy acceptor defects, and V3–In plays a secondary role.^60^

Moreover, researcher found that VIn has many impacts on catalytic activity, optical properties and electronic properties, impacting the material's charge distribution, band structure and charge carrier dynamic properties. Indeed, VIn are associated with deep acceptor behavior, with transition levels in the upper part of the bandgap. They act as charge separation centers, which helps to hinder carrier recombination and enhances the separation and migration of photogenerated charges. This effectively means they can stabilize holes, preventing them recombining with electrons. The surface lone pairs, which are just above the top of the bulk valence band, act as centers for trapping photogenerated holes, placing them optimally for oxidation reactions. Also, VIn plays a crucial role in accelerating specific CO_2_ conversion reaction by creating more active site and improving reaction kinetics. For instance, VIn could enhance CO_2_ adsorption and reducing energy barrier for the formation of the key intermediate *COOH during CO_2_RR.^45,50,61^

In conclusion, while less thermodynamically favorable under typical conditions, VIn provide indispensable electronic assets that complement OVs. By creating deep acceptor states that trap photogenerated holes, VIn efficiently suppresses charge recombination while simultaneously narrowing the bandgap and lowering the energetic barrier for the crucial *COOH intermediate. Having established the fundamental properties of both anion and cation defects, the subsequent section transitions to the advanced characterization techniques required to experimentally identify, verify, and map these intricate vacancy structures.

### 2.3. Importance of vacancy of materials and its applications

OVs can enhance carrier separation, their effect is location-dependent: surface OVs promote electron transfer to the reaction sites, but OVs created in the bulk phase often act as detrimental recombination sites. Consequently, the major hurdle in optimizing In_2_O_3_ performance is the precise, simultaneous regulation of both the concentration and specific location (surface *versus* bulk) of these defects.^17,18,53^ In the other hand, understanding the full mechanism by which the In_2_O_3_ structure governs CO_2_ interaction and product selectivity remains incomplete. While OVs on In_2_O_3_ are widely believed to adsorb and activate CO_2_, the overall catalytic mechanism involving these OVs and other defects needs thorough investigation to precisely define their role in CO_2_ reduction. A significant hurdle is the limited product selectivity observed in reported In_2_O_3_-based systems: they predominantly yield C_1_ gas-phase products (CO, CH_4_) Generating higher value C_1_ liquid-phase products or C_2_ chemical commodities is challenging due to the larger reaction barrier required for both the adsorption/desorption of reaction intermediates and the subsequent intermediate coupling reactions.^18,62^

#### 2.3.1. Light interaction and charge separation

Modifying a material's electronic structure to enhance light absorption often relies on introducing defects, like OVs or metal vacancies. This strategy works by reducing the material's bandgap (*E*_g_). Introducing vacancies creates defect energy levels or mid-gap states within the semiconductor's bandgap, as reported by Wang *et al.* (2024)^63^ and Zhang et colleagues (2023).^64^ These localized states allow electrons to transition using less energy, enabling the material to absorb lower energy photons more efficiently.^15,65–67^

Although light exposure can generate *in situ* surface OVs during photocatalysis to boost light absorption, this phenomenon must be carefully balanced with external vacancy control methods to prevent compromising the structural integrity of the catalyst, indeed, as shown in work of Wang *et al.*, for materials such as In_2_O_3_, light illumination can facilitate the *in situ* formation of new defect sites on the surface.^68^

**Fig. 7:**
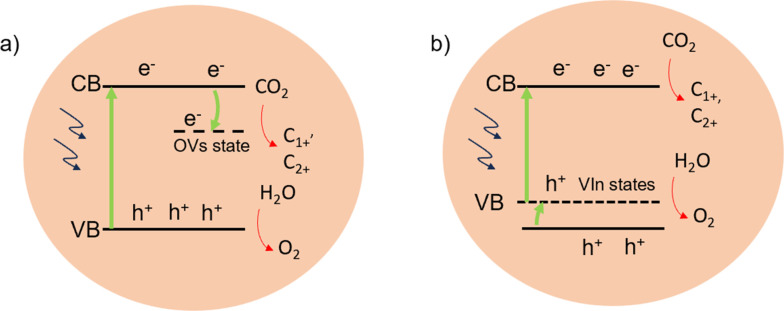
Ligh absorption and recombination (a) over OVs (b) InV.

OVs act like a specialized supercharger for In_2_O_3_'s ability to conduct charge. When light hits the material, it creates electron–hole pairs, which need to be kept separate to do useful work. The OVs sites are excellent at snagging and holding onto the free electrons (acting as “electron traps”) (Fig. 7a).^65–68^ By trapping the electrons, the OVs accomplishes three key things: (i) separate the electron–hole pair,^65^ indeed, electronic modulation induces an upward shift of the Fermi level, lowering the material's work function and binding electrons less tightly. This accelerated separation and migration of photogenerated electron–hole pairs suppresses recombination, while simultaneously shifting the conduction band edge to a more negative potential, ultimately enhancing the material's overall reduction capability; (ii) prolong the life of the excited charge carriers,^15,40,66^ and (iii) improve the material's conductivity and eases charge transfer.^67^ Nevertheless, an excess of defects can actually have a detrimental effect by creating new pathways for the electrons–holes recombination.^40,63,67^ Therefore, finding the optimal balance of OVs concentration within the material is crucial.

Nonetheless, it is important to note that an excess of OVs can induce lattice disorder, thereby diminishing the performance enhancements previously gained.^15,64^

Acting as shallow acceptors, cationic vacancies induce p-type conductivity in semiconductors. Despite requiring higher energy for fabrication compared to anion vacancies, they play a crucial role in enhancing photocatalytic efficiency. Their primary benefit lies in their ability to rapidly segregate and migrate photogenerated charge carriers; this accelerated transfer significantly improves electron–hole separation, thereby driving higher photocatalytic activity (Fig. 7b).^69^

#### 2.3.2. CO_2_ activation and selectivity over oxygen vacancy

To improve charge dynamics, surface OVs play a direct chemical role in the adsorption and activation of CO_2_ molecules, which act as electron rich centers that adsorb and bend the CO_2_ molecule, facilitating charge transfer into its antibonding orbitals.^18,70^ On defective In_2_O_3_ surfaces, CO_2_ binds at OVs sites through In–C coordination and interaction of its oxygen atoms with neighboring indium cations, forming an activated CO_2_^*δ*−^ intermediate. The density and structure of OVs strongly affect this process: appropriate vacancy concentration enhances CO_2_ adsorption and activation, while excessive reduction can destabilize intermediates.^18^

OVs are the crucial, universally accepted active sites in In_2_O_3_ catalysts, acting as the primary engine for various CO_2_RR, including hydrogenation.^58^ Their powerful influence stems from their ability to serve as direct reaction sites, modify the catalyst's electronic properties, and significantly lower energy barriers for key reaction steps, thereby optimizing the thermodynamics and boosting CO_2_ adsorption.^38,40^ In CO_2_ hydrogenation specifically, OVs determine both the overall activity and the selectivity between desirable methanol CH_3_OH and undesirable carbon monoxide CO.^38^ Density Functional Theory (DFT) calculation show that a moderate OVs concentration is optimal because it activates the CO_2_ molecule for conversion to methanol while suppressing the competing CO producing pathway (RWGS reaction).^33^ By fine tuning the vacancy formation energy (ideally between 2.8 eV and 3.3 eV), scientists ensure the methanol forming pathway has a much lower energy barrier, making In_2_O_3_ a highly effective and selective CO_2_ conversion catalyst.^71^ For instance, scholars demonstrated the nonlinear relationship between adsorption process and OVs, noting that the effective adsorption and activation of CO_2_ is governed by the right amount of OVs.^72^

Otherwise, if the OVs concentration is too high, it can be detrimental:^58^ excess surface vacancies make the catalyst prone to over-reduction at high temperatures, potentially forming inactive metallic Indium In^0^ and causing deactivation.^58^ Furthermore, excessive OVs can increase the undesirable competitive adsorption of water H_2_O, a reaction byproduct, which then occupies the active sites and reduces the overall CO_2_ conversion efficiency.^38^

In most previous studies on CO_2_ hydrogenation over oxide catalysts, efforts to enhance activity have primarily focused on modulating the density of OVs. However, Xu *et al.* (2025) introduced a new paradigm by demonstrating that the structure and aggregation of vacancies not merely their abundance, play a decisive role in catalytic performance. By integrating quantum size effects with strong interfacial confinement, they engineered large OVs clusters (such as trimers and tetramers) within monolayer In_2_O_3−*x*_ nano-islands supported on ZrO_2_ using atomic layer deposition techniques (ALD). This configuration stabilizes extended vacancy ensembles that host more localized electrons on adjacent In^*δ*+^ cations, resulting in stronger CO_2_ adsorption and more efficient H_2_ activation. The interfacial confined In_2_O_3−*x*_ remained stable against over-reduction or sintering, and when combined with Pd to facilitate hydrogen spillover. This study thus shifts the focus from vacancy quantity to vacancy architecture, establishing that controlling the size, clustering, and confinement of OVs offers a powerful new approach for optimizing CO_2_ hydrogenation catalysts.^73^

**Fig. 8:**
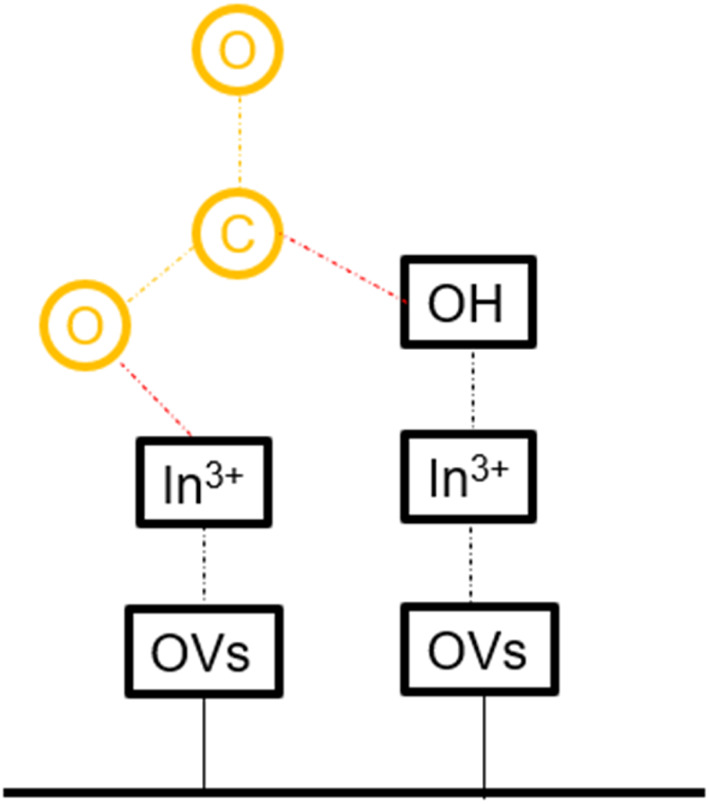
SFLPs effect on In_2_O_3−*x*_(OH)_*y*_ over CO_2_ activation.

Moreover, a related surface feature that frequently coexists with OVs is the formation of SFLPs, which further enhance CO_2_ activation and H_2_ dissociation especially in hydroxylated In_2_O_3_ (In_2_O_3−*x*_(OH)_*y*_) surfaces.^74,75^ A functional SFLP site involves a synergistic arrangement of a Lewis basic hydroxide OH group, an electron-accepting Lewis acidic In atom, and an adjacent OVs^71,75^ as in Fig. 8. SFLPs emerging from adjacent In^3+^-OVs ensembles further promote CO_2_ polarization and H_2_ dissociation, offering a cooperative pathway for activation. Collectively, controlling OVs geometry, aggregation, and SFLP formation now defines the cutting edge in optimizing CO_2_ activation on In_2_O_3_ based catalysts.^53^

Crucially, CO_2_ activation dynamics represent only one aspect of the overall catalytic process; the selectivity toward specific produced species such as CO, CH_3_OH and C_2+_ products are equally vital. In photocatalytic reduction, product selectivity is dictated by a complex interplay of thermodynamic and kinetic factors, including the catalyst's band structure, the nature of its surface active sites, and the specific number of electrons transferred to the adsorbed reactants.^41^ Generally, these influences can be categorized into three core parameters: (i) the electron transfer number, (ii) the configuration of active sites (such as OVs), and (iii) the presence of competitive reactions. Having extensively discussed the latter two parameters in the preceding sections, it is established that optimizing the precise concentration of OVs is mandatory to prevent catalyst deactivation, just as mitigating the competitive HER is essential to maximize CO_2_RR efficiency. To circumvent the detrimental effects associated with these parameters, targeted defect and interfacial engineering strategies offer viable solutions.

CO is a frequently observed product because its formation is kinetically highly favorable and proceeds *via* a straightforward two-electron (2e^−^) reduction pathway. The typical reaction route on the catalyst surface is illustrated below:CO_2_ → *COOH → *CO → CO

High selectivity for CO often exceeding 90% is regularly achieved because it is thermodynamically and kinetically easier for the intermediate to desorb from the surface as a gas rather than remain bound to undergo the complex, multi-electron hydrogenation steps required to yield hydrocarbons. To further steer this pathway, the introduction of dopants such as iron (Fe) or potassium (K) has been shown to significantly enhance CO selectivity. These promotional elements optimize the coordination configurations of the adsorbed intermediates and effectively lower the overall formation energy barrier of the CO product.

On the other hand, methanol (CH_3_OH) production is considerably more challenging to achieve because it requires a complex six-electron (6e^−^) transfer process. Thermodynamically, while the hydrogenation of CO_2_ to methanol is an exothermic reaction meaning lower temperatures favor high equilibrium conversion kinetic barriers typically necessitate thermal activation to drive the reaction forward efficiently. Mechanistically, the formation of the methoxide (*CH_3_O) intermediate represents a critical, rate-determining step along the pathway to methanol. To overcome these kinetic bottlenecks, recent studies have focused on engineering indium oxide to create SFLPs. These specialized sites heterolytically cleave H_2_ into a reducing hydride and an acidic proton, a strategy that has been shown to yield the highest selectivity toward methanol (Fig. 8).^76,77^

Overall, while OVs are widely celebrated as essential active sites that lower the activation energy for CO_2_ adsorption and intermediate stabilization,^78^ they inherently act as a double-edged sword. As discussed, maximizing OV concentration does not linearly correlate with enhanced CO_2_ reduction performance. Instead, researchers must find a strict optimal threshold that balances active site density with charge transfer efficiency, while carefully restricting OVs to the catalyst surface. Failure to control this architecture not only quenches catalytic efficiency *via* charge recombination but also drives severe catalyst deactivation through irreversible over-reduction to metallic indium.^79^

Furthermore, the selectivity regarding multi-carbon (C_2+_) products presents a significant challenge due to the high kinetic barriers associated with the C–C coupling step. Notably, there are no existing studies reporting pristine indium oxide as the primary catalyst for standalone C_2+_ production. Instead, insights from alternative catalytic systems indicate that maximizing C_2+_ selectivity requires a multi-functional design strategy. The most effective configurations frequently utilize S-scheme or Z-scheme heterojunctions, or specialized co-catalysts, to mitigate rapid charge carrier recombination. Concurrently, the intentional introduction of OVs is employed to extend light absorption, while the integration of plasmonic metals helps generate highly energetic hot electrons. Finally, these elements are often coupled within a tandem framework such as a porous matrix which serves to confine intermediates and lower the activation energy required for the pivotal C–C coupling step.^80^

Therefore, future engineering strategies must move beyond merely increasing OV quantity. The next frontier in In_2_O_3_ catalyst design relies on precise defect localization, controlling vacancy architecture (such as size and clustering), and ensuring long-term structural stabilization.

### 2.4. Control for vacancy engineered In_2_O_3_

#### 2.4.1. Thermal treatment

The precise synthesis and control of OVs are primarily achieved through two key mechanisms: thermal under atmospheric manipulation and chemical reduction strategies. Thermal annealing is the foundational method, where high temperatures combined with a reducing gas like hydrogen H_2_ are highly efficient, as the gas acts as an oxygen scavenger, drastically lowering the required processing temperature and time compared to simple air calcination. The second mechanism, chemical reduction, involves introducing solid additives such as oxygen-affine materials like alumina or a carbon source during calcination. These materials work indirectly by effectively lowering the local partial pressure of oxygen P(O_2_), thereby promoting OVs formation, a mechanism that is also influenced by controlled doping elements that modify the local chemical and electronic environment of the crystal structure. Table 5 gives some most recent work about thermal annealing.

**Table 5:** Summarize of recent work about thermal annealing over In_2_O_3_

Temp. (°C)	Atmosphere	OVs trend/observation	Catalytic effect on CO_2_ reduction	Ref.
400–800	Air	High temperature increases OVs density and switch from rods to porous	Moderate OVs (600 °C) optimize surface area & activity	81
250–450	Air	High temperature led to stronger OVs surface	None	82
250	Air	Morphology dictates OV density (nanocrystals *vs.* rods)	Active sites for CO_2_ hydrogenation due to SFLP formation (between In and hydroxide group)	77
550	H_2_/He (5%)	10 minutes H_2_ treatment led to bulk OVs whereas longer time led to surface OVs	Controlled hydrogenation time tunes OVs distribution	83
300	5%H_2_/Ar air	H_2_ rich-sample led to highest OVs concentration	Enhances CO_2_-adsorption, lowers CO desorption energy (facilitate release from the catalyst)	84
350	H_2_	h/c-In_2_O_3_ structure led to highest OVs concentration OVs at lower temperatures and in larger quantities do not necessarily enhance activity	h/c mixture phases induced effective CO_2_ activation whereas c-In_2_O_3_ has superior stability and regeneration during CO_2_RR	85
400	Ar induce surface oxygen defect then 73/24/3 (H_2_/CO_2_/N_2_) for CO_2_RR	RWGS resulted in an in-*operando* production of OVs due to CO molecule	CO_2_ chemisorbs at the OVs site to form 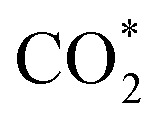 to methanol. The introduction of CO into the feed gas boosts the CO_2_ hydrogenation activity to C_5+_	86
220–300	72/24/4 (H_2_/CO_2_/N_2_)	Surface OVs generation H_2_ induces OVs at activation	OVs promote CO_2_ adsorption/activation. Favoring CH_3_OH	87
250–350	3/1/1 (H_2_/CO_2_/N_2_)	The quantity of OVs depend on the temperature and hydrogen partial pressure	Harsh operating condition (T, P, gas ratio) lead to catalyst deactivation CH_3_OH exhibits better selectivity in the rhombohedral phase	88
250–400	Al-assisted reduction	Al scavenges O resulted in surface OVs which also increase with temperature	None	37
350–700	Ar/air	C removes lattice O in order to tune OVs density	Controlled OVs density maximizes CH_3_OH formation	38
300–400–500	H_2_/Ar (5%) Ni co-catalyst	OVs enhanced visible light absorption Ni NPs boost light-harvesting	The Ni loading on In_2_O_3−*x*_ can decrease the energy barrier for CO_2_ conversion. Ni lower the reduction temperature	89
350	3/1 (H_2_/CO_2_)	GO promotes the formation of OVs at the interface of the c/h In_2_O_3_ phase homojunction	Production of CH_3_OH: the In_2_O_3_-8 wt% GO catalyst, which has the highest concentration of OVs, exhibits the best performance	90

#### 2.4.2. Doping

**Fig. 9:**
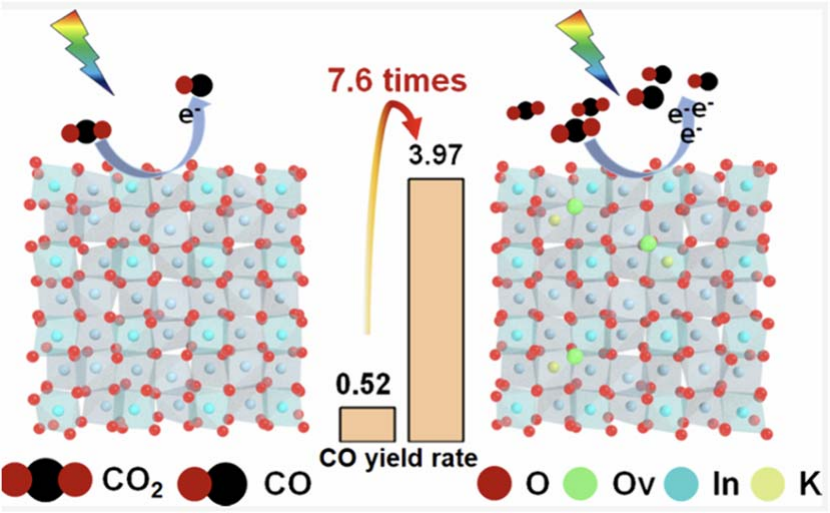
Effect on N-doping over CO_2_ photoreduction, reproduced from ref. 93 with permission from American Chemical Society,^93^ copyright 2024.

Beyond thermal strategies, doping provides another powerful lever for tuning OVs concentration. For instance, Thang *et al.* utilized Fe single atoms to tailor oxygen vacancies within the Bi_2_WO_6_ photocatalyst. The Fe atoms may substitute for W atoms due to their similar ionic radii. Because of the difference in their oxidation states, the lattice compensates by creating additional oxygen vacancies to maintain electroneutrality. This modification optimizes the band structure for broader light absorption, provides more active sites, and reduces the reaction's activation energy.^91^ Doping approach is also effective for controlling over-reduction and avoid catalyst deactivation. Indeed, an excessive number of OVs on the surface of an In_2_O_3_ catalyst, especially at high temperatures, can lead to its over-reduction, resulting in an amorphous, less effective surface.^92^ This surplus of vacancies also acts as a site for electron–hole recombination, which disrupts the charge transfer necessary for photocatalytic reactions.^40^ To optimize these vacancy levels, researchers have introduced various dopants. This method also improves the material's electronic properties, which in turn enhances light absorption and reactant activation.^17,18^ By substituting certain atoms within the crystal lattice (Fig. 9), doping can induce lattice distortion or lattice mismatch due to the different ionic radii or valences leading to structural strain, which in turn promotes the formation of OVs. Another method involves exploiting the aliovalent effect of dopants on indium atoms, where an ion with a different charge substitute for a host ion (In^3+^). This charge imbalance necessitates the ejection of oxygen to restore the crystal's electrical neutrality, creating OVs. Aliovalent dopant effectively controls the concentration of these vacancies, thereby improving the material's stability and performance.

Several recent studies presented in Table 6 demonstrate these principles using various dopants.

**Table 6:** Summarize of recent work about doping over In_2_O_3_

Dopant system	Mechanism for OVs formation	Observed structural/Electronic effect	Ref.
Bi	Synergistic effect between larger ionic radius and lone pair weakened Bi–O bond leading to O removal	OVs density is the highest near Bi sites after calcination	94
K	Aliovalent doping (K^+^/In^3+^) induces a charge imbalance, which trigger a Jahn–Teller distortion	Bond weakening	95
Fe	The two ways of formation are the reducible atmosphere and FeO_*x*_ modulation under UV	Fe–O–In interface ensure electron transfer channel during photocatalysis	40
N	Difference of radii with O atoms ⇒ lattice distortion surface OVs	Band gap narrowing enhanced charge separation stability	14
ZrO_2_ support	Interface lattice mismatch allows O diffusion at boundary	ALD deposition gain 30% surface OVs *vs.* IWI	96

#### 2.4.3. Other methods

In recent, novel physical or chemical approaches have been proposed to achieve even finer control of OVs in In_2_O_3_. These have important implications for applications in catalysis and gas sensing.

As a physical route, Jung *et al.* (2023) introduced a new method for controlling the amount of oxygen on the surface of In_2_O_3_ semiconductors. They accomplished this using a microheater transistor that applies an electric field at a high temperature of 240 °C. This electric field directly influences the MO_*x*_'s electron concentration, which, in turn, changes the energy required for oxygen to bind to the surface. The core of their approach is a direct relationship between applied voltage and electron concentration; for instance, a positive voltage increases the electron concentration, promoting oxygen adsorption and making the surface O-rich, which also reduces the number of OVs. On the other hand, a negative voltage decreases the electron concentration, which causes oxygen desorption to occur. By using this method, the researchers demonstrated a reconfigurable way to control surface oxygen on MO_*x*_ materials, which they implemented by fabricating thin film transistors with embedded microheaters.^97^ In a different vein, Ze *et al.* (2023) synthesized OVs-rich indium oxyhydroxide (InOOH-OVs) nanosheets by treating standard InOOH with a plasma reduction method under an Ar atmosphere. This technique selectively stripped surface lattice oxygen, creating OVs which manifest as lattice distortion and charge redistribution within the material. These OVs are vital to the material's performance: the resulting charge redistribution enhances the adsorption and activation of CO_2_ molecules, and the OVs also prevent the indium from being over-reduced during the catalytic cycle. This robust, OVs-rich material functions as a highly effective bifunctional catalyst for the CO_2_RR, notably boosting the FE of formate production.^56^

**Fig. 10:**
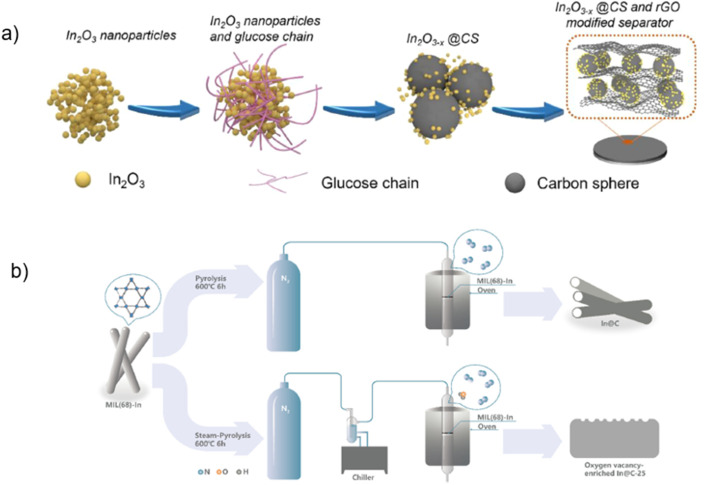
(a) Schematic fabrication of In_2_O_3_-rich OVs using glucose as precursors, reproduced from ref. 98 with permission from Elsevier,^98^ copyright 2022. (b) Schematic illustration of the stream-pyrolysis and pyrolysis process, reproduced from ref. 99 with permission from Royal Chemical Society under the terms of the Creative Commons Attribution License,^99^ copyright 2025.

In contrast, as a chemical route, Zou *et al.* (2022) created OVs-rich In_2_O_3_ nanoparticles using a hydrothermal method. They used glucose as a reducing agent, varying the amount of glucose (80, 100, 120, and 140 mg) to control the number of OVs (Fig. 10a). During the process, the glucose polymerizes and pulls unstable oxygen atoms away from the In_2_O_3_, thereby increasing the oxygen defects on the surface. Consequently, using more glucose results in a higher concentration of OVs in the nanoparticles.^98^ Alternatively, Wang and colleagues (2025) synthesized the In_2_O_3_ MOF, MIF(In)-68, and utilized a strategic two-stage heat treatment initial calcination at 600 °C followed by water-steam pyrolysis in an N_2_ atmosphere to engineer OVs (Fig. 10b). The researchers precisely controlled the number of OVs by adjusting the steam partial pressure (water content). Mechanistically, the introduction of these OVs plays a pivotal role in boosting the catalyst's performance in reactions like the RWGS.^99^

On the whole, it is worth recalling that, controlling the concentration of OVs is crucial for effective catalysis. An optimal amount of OVs can enhance catalytic performance, but too many can be detrimental. In thermocatalysis, an excess of OVs can lead to overly strong CO_2_ adsorption, which hinders the catalytic cycle. Similarly, in photocatalysis, an abundance of these vacancies can create electron–hole recombination centers, reducing the overall efficiency. Consequently, rational defect engineering is used to precisely tune the concentration of these vacancies to achieve the best possible catalytic outcome.^100^

In summary, the deliberate engineering of oxygen vacancies in In_2_O_3_ has evolved from simple thermal treatments to a sophisticated toolkit of synthetic strategies, including precise aliovalent doping, plasma-assisted reduction, and advanced interfacial confinement techniques. These methodologies have fundamentally shifted the field's focus: instead of merely increasing raw vacancy density, current approaches prioritize the architectural control of vacancy clusters and their spatial distribution. By moving toward these tailored, site-specific engineering paradigms, researchers can now optimize the electronic environment of In_2_O_3_ enhancing light-harvesting, charge separation, and reactant activation while simultaneously mitigating the structural instabilities that previously limited catalyst durability. Ultimately, the ability to synthesize these precisely defined vacancy architectures provides the foundation for the next generation of highly active and stable catalysts. Therefore, the subsequent section details the advanced characterization techniques required to verify these engineered structures and confirm the successful identification of vacancy-engineered In_2_O_3_.

### 2.5. Identification of vacancy engineered In_2_O_3_

The characterization of materials is a fundamental step in materials science, essential for understanding the intrinsic properties, defect structures, and ultimately, the performance of advanced functional materials like metal oxides. A particular focus in modern research is the identification and quantification of OVs, which are point defects that profoundly influence a material's electronic structure, chemical reactivity, and catalytic activity in applications such as gas sensing and photocatalysis. To accurately probe these critical defects, researchers rely on a suite of complementary spectroscopic and microscopic techniques, each offering unique insights from detailed surface chemistry to bulk defect concentrations and electronic structure modification. The following sections will explore several key techniques, including XPS, Raman, EPR, PL spectroscopy, EIS, H2-TPR and electron microscopy. Table 7 detail their specific mechanisms and how they are applied to successfully monitor and analyze OVs in materials such as In_2_O_3_.

**Table 7:** Most use characterization techniques to control vacancies over In_2_O_3_

Technique	Typical signature of OVs	Insights for CO_2_ photoreduction	Methods
Main principle			
XPS	Characteristic peak between 530.7 and 531.4 eV (ref. 37, 38, 82, 84, 92 and 98)	Quantifies surface OVs density^37,38^	Relative peak area ratio/total area of all O peak^38,92^
Surface-sensitive probe of oxidation states and bonding	In 3d_5/2_ shift to lower binding energy with increasing OVs^98,101^	Tracks valence changes under doping/annealing^82,97^	Concentration of Ovs differ from authors^102–104^
			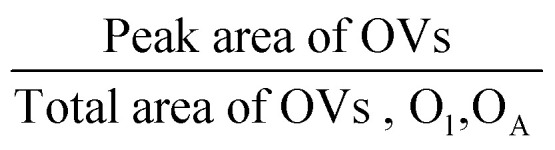
			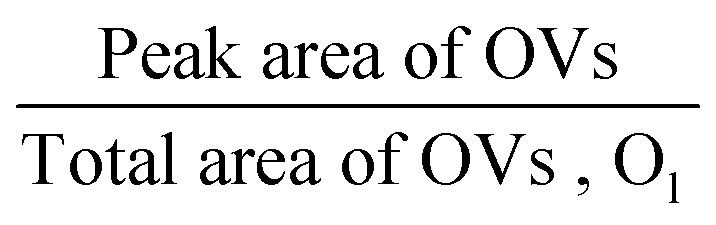
			(Peak area of OVs)/(Total area of In–O)
			O_l_: lattice oxygen
			O_A_: adsorbed species
Raman	Characteristic peak	Can use in-*operando* to identify intermediate molecule during CO_2_RR	Concentration of OVs differ from authors^87,90^
Vibration modes of material	304–306 cm^−1^ (InO_6_)^92^		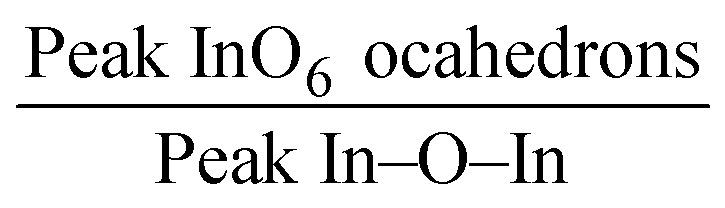
	364–366 cm^−1^ (In–O–In)		
EPR	Characteristic signal *g* ≈ 2.001–2.003 (OVs^+^)	Directly quantifies active OVs and monitors dynamic regeneration under light^34,41^	Intensity of EPR signal is directly proportional of OVs amount^37,106^
Detects unpaired electrons (paramagnetic defect centers)	EPR-silent: (OVs^0^, OVs^2+^)^34,84,98,105,106^		
PL	Characteristic peaks	lower PL intensity implies better separation^37,95^	PL signal is proportional of OVs density^37,106^
Monitor radiative recombination of e^−^/h^+^ pairs	Broad emission: ∼530 nm (OVs^0^)^45^		
	∼470 nm (OVs^+^)^106^		
EIS	Comparative methods^63,107^	The smallest charge transfer resistance promotes the kinetic process to form formic acid^107^	The smaller semicircle radii, the higher OVs concentration^107,108^
Electron transport properties			
HRTEM, EELS	Surface O–K edge shift (*e.g.*, 537.5 to 532 eV)	Lattice expansion near surface indicate better catalytic activity^109^	The larger shift on the surface indicates a higher concentration of OVs^109^
Atomic scale imaging of lattice, interfaces, and strain	Bulk O–K edge shift (*e.g.*, 537 to 535 eV)^109^		
H_2_-TPR monitors H_2_ concentration	OVs surface: 130 °C to 350 °C	The area under these peaks is used to quantify the amount of hydrogen consumed^38,90^	High H_2_ consumption often indicates low initial vacancies^76^
Decrease mean reduction	Extraction of surface lattice oxygen^90,103^		
	OVs bulk: above 400 °C		
	Deep reduction of the internal lattice^99^		
	H_2_ + O_lattice_ → H_2_O + OVs		
DFT & combined simulations (DFT + ML, BRANNs)	Predicts defect-induced bandgap narrowing, charge redistribution^61^	Correlates defect configuration with optical and catalytic performance^61^	None
Computes formation and adsorption energies, electronic states			

A major limitation in defect characterization is that conventional techniques such as Raman spectroscopy, PL, and XPS cannot distinctly differentiate between bulk and surface OVs. XPS is inherently confined of the material's surface, making it blind to deep bulk states, whereas Raman and PL lasers penetrate deep into the bulk matrix, thereby capturing an average signal that indiscriminately blends surface and bulk contributions. To isolate these spatial distributions, specialized techniques with unique signal sensitivities must be deployed, most notably EELS and H_2_-TPR. However, specialized techniques such as EELS and H_2_-TPR are rarely utilized for routine quantification in the broader literature due to the high cost and limited instrument availability associated with EELS, alongside the destructive thermal nature of H_2_-TPR. Instead, the vast majority of published studies rely on XPS and Raman spectroscopy as the primary, accessible standards for defect quantification. To highlight these trends, Table 8 compiles the key literature results regarding OVs concentrations as quantified by XPS and/or Raman spectroscopy.

**Table 8:** OVs concentration measured by XPS or Raman

Material	Optimum OVs concentration	Reaction type	Way of measurement	Ref.
OVs-In_2_O_3_	20%	Photothermal	XPS: peak area ratio of (∼530.7 eV) to total oxygen species	110
OVs-In_2_O_3_	28.74%	Electrochemical	XPS: peak area ratio of the oxygen vacancy (∼531.7 eV) to In–O bond (∼529.8 eV)	104
0.5%-Ru/In_2_O_3_	44.2%	Dehalogenation	XPS: ratio of peak area at ∼530.9 eV to all oxygen species	111
MOF-derived In_2_O_3_@C	35.3%	Photothermal	XPS: relative contribution of component at 530.7 eV (oxygen defects)	112
			Raman: peak intensity ratio between unsaturated (357 cm^−1^) and saturated (126 cm^−1^)	
In_2_O_3_GO	53%	Photothermal	XPS: ratio of peak area at ∼530.9 eV (*A*_Odefect_/(*A*_Olattice_ + *A*_Odefect_ + A_OOH_)) to all oxygen species	113
In_2_O_3_/CdSe-DETA	30%	CO_2_RR	XPS: peak area ratio of chemisorbed oxygen (∼531.13 eV) caused by vacancies	114
Pd–P/In_2_O_3_	63.4%	Photothermal	XPS: relative concentration formula: *A*_defect_/*A*_defect_ + *A*_Olattice_	103
			Raman: intensity increase of 364 cm^−1^ (In–O–In) and 130 cm^−1^ (In–O vibration)	

A cross-examination of the literature reveals a profound lack of consensus regarding what constitutes the “optimal” concentration of OVs in In_2_O_3_ frameworks. Across diverse applications including CO_2_RR, photothermal catalysis, dehalogenation, and electrochemical systems reported optimal vacancy thresholds are heavily scattered, ranging from 20% to as high as 63.4% (as summarize in Table 8). This variance demonstrates that the “optimum” is highly dictated by fundamental discrepancies in data calculation methods across different research groups, particularly in X-ray Photoelectron Spectroscopy (XPS).

While the binding energy assignments for the O_1s_ XPS envelope in In_2_O_3_ are well-established (O_lattice_ at 529.8–530 eV, OVs at 530.7–531.7 eV, and O_OH_ at 532 eV), the mathematical formulas applied to extract concentrations vary drastically:

• Total oxygen reference: calculating the peak area ratio of OVs relative to all oxygen species (*A*_ov_/*A*_total_) yields optimal values of 20% for photothermal reactions and 44.2% for dehalogenation (0.5%-Ru/In_2_O_3_).

• Lattice cross-referencing: conversely, referencing the vacancy peak strictly against the lattice framework such as the ratio *A*_ov_/*A*_In–O_ (yielding an electrochemical optimum of 28.74%) or the relative contribution formula *A*_ov_/(*A*_ov_ + *A*_lattice_) (yielding 63.4% for Pd–P/In_2_O_3_) drastically shifts the reported percentages.

In contrast to the mathematical inconsistencies found in XPS deconvolution, Raman spectroscopy demonstrates much better coordination and consistency across different papers. Authors exhibit a strong consensus regarding both peak positioning and intensity ratio evaluations to track physical lattice disorder. For instance, structural distortions are reliably tracked *via* intensity ratios between unsaturated and saturated lattice modes (such as the 357/126 cm^−1^ ratio at 35.3% OVs) or *via* the characteristic red-shift of the external InO_6_ octahedral mode at 130 cm^−1^ accompanied by changes in the In–O–In stretching vibration at 364 cm^−1^.

Overall, the significant variance in reported ‘optimal’ vacancy concentrations underscores that simply creating defects is not enough; the true challenge lies in maximizing their functional utility. Because different applications demand distinct electronic and chemical behaviors, modern research focuses heavily on unlocking and amplifying the precise benefits that these defects provide such as accelerated charge transport, enhanced adsorption kinetics, and tuned bandgaps. To move beyond basic defect creation and toward true performance optimization, the next section explores the strategies to enhance vacancy engineered In_2_O_3_, detailing how researchers stabilize, activate, and amplify the catalytic and electronic advantages of these vital structural defects.

### 2.6. Strategies to enhance vacancy engineered In_2_O_3_

The introduction of OVs in In_2_O_3_ catalysts has been shown to improve performance by increasing the absorption of visible light, promoting the separation of photogenerated electrons and holes, and enhancing the activation of reactant molecules like CO_2_. Nevertheless, the catalytic performance of OVs engineered In_2_O_3_ may be significantly amplified through synergistic strategies such as morphology control, heterojunction but also cocatalyst strategies. These combined approaches are key to overcoming the limitations of single-strategy modification and are essential for developing highly efficient and selective In_2_O_3_-based catalysts. The following sections highlight three synergistic design strategies morphological control, heterojunctions and cocatalysts that further amplify the benefits of vacancy engineering.

#### 2.6.1. Morphology control

Morphology control is a crucial strategy to expose a high density of OVs on specific crystal facets, optimizing the catalyst's surface area and accessibility of active sites for enhanced performance.^53^ The enhancement of OVs concentration through morphology control is often observed as a synergistic effect resulting from specific synthetic strategies that simultaneously regulate both the material structure and its defect content. Morphology control may influence OVs concentration through correlation with crystallinity, surface area, or deliberate manipulation of growth conditions that yield both a specific shape and defect content.

For instance, Mukherjee *et al.* (2018) used a vapor–liquid–solid method to growth In_2_O_3_ structure with two morphologies (nanowires and octahedrons nanoparticles) using different source material. They have demonstrated that by controlling the growth conditions (deposition zone temperature, oxygen partial pressure or carrier gas flow rate), they promote specific facets orientation. Therefore, scholars can influence the concentration and location of surface OVs.^60^

**Fig. 11:**
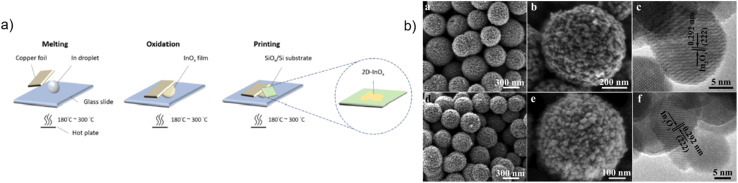
(a) Process flow demonstrating fabrication of 2D-InO_*x*_ via liquid metal printing, reproduced from ref. 115 with permission from American Chemical Society under the terms of the Creative Commons Attribution 4.0 International License,^115^ copyright 2024. (b) SEM and HR-TEM image of porous In_2_O_3_ and OVs-In_2_O_3_ nanospheres, reproduced from ref. 37 with permission from Elsevier,^37^ copyright 2020.

In a different note, Chen *et al.* (2024) have shown that controlling grain boundaries is crucial because they significantly influence the material's properties by acting as preferential sites for the accumulation of defects, particularly OVs (Fig. 11a). Since OVs are concentrated at the grain boundaries, these boundaries become the critical pathways and zones that govern the electrical performance, therefore, by controlling the density and distribution of grain boundaries, one can directly manipulate the functional properties of the material. Researchers have noted that higher temperature increases the grains size and simultaneously decreases the overall concentration of OVs in the material.^115^

##### 2.6.1.1. Designing porous structure

Porous structures are crucial for boosting the effectiveness and stability of OVs in MO_*x*_ catalysts, creating an ideal environment for them to function. These structures, including dendritic forms, nanowires, and hollow microcubes, dramatically increase the catalyst's surface area, which in turn boosts the density of highly active surface OVs over less-effective bulk OVs. This higher surface area and the intricate network of pores also improve the adsorption and mass transfer of molecules, ensuring reactants efficiently reach the active sites. While these robust, porous morphologies enhance long-term stability, significant challenges remain in precisely controlling OVs, and our understanding of their dynamic behavior and full photocatalytic mechanism is still incomplete.^17,18,53,62^

Morphologies with a high surface-area-to-volume ratio, such as hollow nanostructures or porous materials, can enhance the effects of OVs. Since OVs are often concentrated on the surface of nanowires and other nanostructures, increasing the specific surface area exposes more of these defect-rich sites. Moreover, that kind of structure provide a large number of active sites for reactions, improve the adsorption of reactants like CO_2_, and enhance light utilization through scattering and reflection effects. The shell structure also shortens the diffusion path for charges. For example, several scholars have shown that hollow structures as well as porous or high specific area structures enhance gas molecule adsorption by optimizing the impact of OVs.^116–118^

The larger the surface area, the more readily OVs can form. For instance, porous nanostructured In_2_O_3_ has a greater specific surface area than its bulk form, leading to a higher concentration of OVs. For example, by controlling these defects, its magnetic properties can be enhanced.^119^ Nevertheless, an overly high concentration of OVs can lead to structural changes that hinder performance. For example, in porous In_2_O_3_ nanospheres (Fig. 11b) used for gas sensing, too many OVs can lower electron mobility. This happens because the escape of oxygen from the In_2_O_3_ surface creates a disordered crystal structure, which in turn forms a high energy barrier at the grain interfaces, making it harder for electrons to move. This illustrates a direct link between the physical arrangement of OVs and a loss of catalytic performance.^37^ To prevent a catalyst from deactivating and to improve its durability, it's essential to both stabilize OVs and maintain their concentration at an optimal level. For instance, in porous, single-crystalline beta-Gallium Oxide (β-Ga_2_O_3_) monoliths, the highly ordered crystal structure helps keep OVs stable. This prevents degradation and boosts catalytic activity and durability, even during extended use.^36^

##### 2.6.1.2. Designing bridging oxygen vacancy

A significant challenge in CO_2_ reduction is preventing OVs from being refilled by oxygen atoms from CO_2_ molecules, which deactivates the catalyst. Bridging OVs can help overcome this problem. Designing bridging OVs (one oxygen atom between two metal atoms) is particularly important because they can greatly improve the performance of photocatalytic materials, especially for applications such as CO_2_ reduction. This kind of configuration offers a range of advantages. It enhances the adsorption and activation of CO_2_ by enabling a “bidentate” bond, where a single CO_2_ molecule attaches to two adjacent metal atoms at once. This stronger, more stable interaction is crucial for activating the molecule. A critical benefit of this bridging structure is its ability to prevent the OVs from being consumed by oxygen atoms from the CO_2_ molecules, which is key for long term durability. Furthermore, like other OVs, these bridging vacancies create new energy levels that can trap electrons generated by light. This effectively lowers the energy barrier for the photocatalytic reaction, making it much easier to activate the CO_2_ and convert it into valuable products. For example, by applying a reduction method, Zhao *et al.* successfully created bridging OVs in the SnTaO_6_ structure. This bridging structure creates highly stable OVs without the need for an additional co-catalyst, which also boosts the overall photocatalytic performance.^120^ Moreover, A study by Mao *et al.* (2024) demonstrated that applying a reduction potential to a tin oxide catalyst surface creates bridging OVs, which are crucial for the efficient conversion of CO_2_ to format (HCOOH). These specific vacancies are not just missing oxygen atoms; they create a unique Sn-OVs-Sn structure that is key to the catalyst's function. The research found that a moderate number of these vacancies is optimal because they improve the binding of intermediate molecules necessary for the reaction. If the catalyst surface is too reduced and has too many vacancies, it becomes too inert, causing the intermediates to bind too weakly, which ultimately lowers the catalyst's activity.^121^

One of the key properties of bridging OVs is that they act as an intermediate for transferring electrons. For instance, researchers have demonstrated that OVs situated between two lattice cations induce a change in the cations' valence states to maintain charge equilibrium. This electronic transition subsequently facilitates electron transfer through an established channel. This change in valence state enhances the material's catalytic properties, despite the high stability of the bridges during catalytic reactions.^122,123^

#### 2.6.2. Heterojunctions

Creating heterojunctions between two different semiconductors can increase the number of OVs.^39,124^ Whilst various heterojunction techniques have been studied to enhance the effectiveness of these OVs, we'll first focus on S- and Z-scheme junctions, which have received more recent attention in research and then focus on P–N junctions. A key feature of these heterojunctions is the creation of a built-in electric field, which significantly boosts electron transfer properties.

The first interest is that vacancy inside a heterojunction directly dictated the underlying charge transfer mechanism. For instance, Sun *et al.* have developed a Bi_2_S_3−*x*_@Cd_0.7_Zn_0.3_S heterojunction where the concentration of sulfur vacancies (Sv) is modulated. A key insight of this work is the ability to tune photoelectron transfer pathways by adjusting vacancy levels.^125^

Also, research has increasingly focused on the synergistic effects between Z-scheme heterojunctions and OVs to improve charge transfer and reactivity. For instance, researchers have highlighted that Z-junction enhances the OVs presence, optimizes the charge transfer and redistribute charge around the OVs, allowing them to facilitate charge migration rather than act as recombination traps.^126,127^ However, further work is still needed to improve the interface to reduce recombination and shorten the charge transfer pathways.

**Fig. 12:**
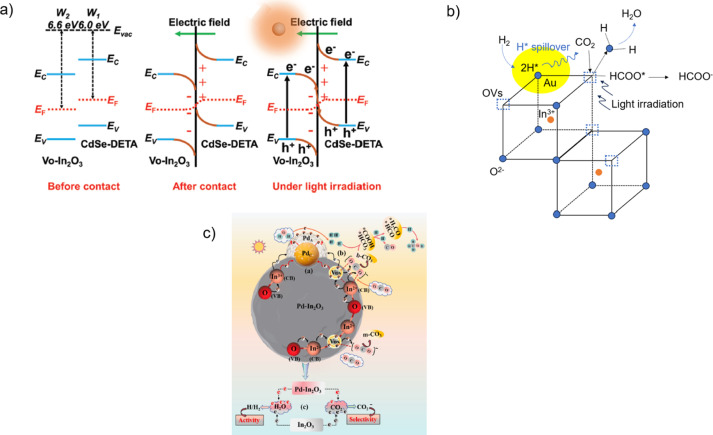
(a) S-scheme heterojunction of OVs-In_2_O_3_ and CdSe-DETA, reproduced from ref. 76 with permission from Wiley,^76^ copyright 2023. (b) Effect of gold co-catalyst and spill-over effect on indium oxide over CO_2_RR (c) Mechanism of photocatalytic CO_2_ + H_2_O reduction over Pd–In_2_O_3_, from ref. 41 with permission from Elsevier,^41^ copyright 2024.

Although the Z-scheme is a well-established concept, the S-scheme heterojunction has recently emerged as a highly effective alternative. These structures (Fig. 12a), leverage OVs as a crucial intermediary for electron transfer through synergistic effects. This unique mechanism can precisely control electron pathway, shorten the transfer distance to the catalyst surface and thus improve light separation.^76,108^

Along the same lines, Wang *et al.* demonstrated that creating a p–n heterojunction between Fe_*x*_O_*y*_ and In_2_O_3_ significantly improves the efficiency of CO_2_ reduction. This enhancement is largely due to the synergistic effect with OVs. The formation of a seamless Fe–O–In interface between the FeO (100) and In_2_O_3_ (222) surfaces and the Fe_2_O_3_ (104) and In_2_O_3_ (400) surfaces establishes an efficient pathway for electron transfer. The number of OVs is also increased through a “refill effect” from the Fe_*x*_O_*y*_, which helps optimize the system and prevent the vacancies from becoming recombination centers. This heterojunction design also shifts the conduction band to a more negative position, resulting in a stronger reducing ability. Furthermore, the researchers found that In^2+^, Fe^2+^, and the OVs work together as active sites, acting as electron donors to pre-reduce CO_2_ and H_2_O molecules and, thereby, boosting catalytic activity.^40^

A notable application of heterojunctions is their ability to prevent the over-reduction of In_2_O_3_ during the creation of OVs, a process that often leads to the formation of metallic indium (In^0^). Research by Wang *et al.* (2024) demonstrated that highly dispersed silica species (SiO_*x*_) on the In_2_O_3_ surface form stable indium-silica bonds (In–O–Si). These bonds are created when methoxysilane reacts with the hydroxyl groups on the In_2_O_3_ surface, and they effectively hinder the conversion of indium species to their metallic form. This stabilizing effect of the SiO_*x*_ heterojunction helps preserve the indium in a more oxidized state, which in turn leads to superior structural stability, reduces catalyst deactivation, and enhances catalytic performance by three orders of magnitude.^128^ While heterojunctions primarily enhance charge separation, cocatalysts complement them by facilitating surface reactions and vacancy regeneration.

#### 2.6.3. Cocatalysts

The advantages of co-catalyst primarily stem from their ability to create synergistic effects that overcome the limitations of single-component catalysts.^62^ The primary advantages of using a co-catalyst are the dissociation of hydrogen and the overcoming of overreduction limitations. Co-catalysts like gold (Au), palladium (Pd), and nickel (Ni) are highly effective at dissociating hydrogen gas (H_2_) when they are decorated onto an In_2_O_3_ catalyst. This process produces hydrogen adatoms (H adatoms) which can then spill over to the support, facilitating the formation and regeneration of surface OVs that are crucial for hydrogenation reactions. For example, Hou *et al.* (2023) demonstrated that the combined effect of an Au co-catalyst improves overall catalytic performance due to the strong interaction between Au and In_2_O_3_ leading to the formation of surface OVs, which aid in CO_2_ adsorption and activation to formate (Fig. 12b).^129^

To mitigate the risk of overreduction in catalysts with strong co-catalyst interactions, recent research has explored the use of dual co-catalyst systems to increase the OVs concentration. For instance, Huang *et al.* (2025) have modulated the lattice strain with Ni and Cu cocatalyst leading to increase the OVs concentration.^130^ Also, Jin *et al.* (2025) have demonstrated that by introducing a Cu–Co alloy, researchers leveraged the strong interaction between the metals to promote H_2_ production and subsequently increase the concentration of OVs.^131^

Another effect of adding a co-catalyst to an In_2_O_3_ catalyst is the precise control of electron transfer, which allows for the regulation of the CO_2_RR. Wang *et al.* (2024) demonstrated this using a hierarchical PdA/PdC/In_2_O_3_ structure, in which electrons migrated sequentially from In_2_O_3_ to crystalline Pd (PdC) and finally to amorphous Pd (PdA). Acting as an electron pump, PdA accumulated electrons on its surface, while the interaction between PdC and OVs partially withdrew electrons from the OVs, reducing their electron-donating capability toward adsorbed CO_2_. This moderated electron transfer suppressed excessive CO_2_ reduction, thereby favoring the selective formation of CO and CH_3_OH (Fig. 12c). Consequently, the Pd-modified catalyst exhibited a 4.5-fold enhancement in CO_2_ reduction activity and a 3.2-fold increase in CO + CH_3_OH selectivity (63.62%) compared with pristine In_2_O_3_^41^. In a separate study, Mao *et al.* (2024) showed that decorating In_2_O_3_ with copper oxide (CuO) to form a heterojunction significantly enhanced catalytic performance due to a strong electronic interaction arising from the difference in their work functions.^85^ This electronic modulation optimized the catalyst's electronic structure, which in turn regulated the adsorption/desorption strengths of reactants and intermediates, exposing more active sites. The formation of these heterogeneous interfaces also generated abundant OVs due to lattice mismatches between CuO and In_2_O_3_. The synergy of the heterojunction and OVs ultimately increased the DOS near the *E*_f_, improving electronic conductivity and electron transfer capabilities.^132^ Also, according to Wang *et al.* (2018), modifying In_2_O_3_ with a Pt co-catalyst alters its surface chemistry and electron behavior. Due to its high electronegativity, Pt pulls electron density away from the In_2_O_3_ surface, increasing chemical binding energies. Furthermore, the Pt co-catalyst optimizes electron flow by forming a Schottky junction which prevents rapid electron–hole recombination and by serving as a localized atomic hydrogen reservoir. This abundant supply of hydrogen adatoms enables efficient carbon-hydrogen bonding on the Pt surface. Consequently, the catalyst shifts the reaction mechanism from a simple two-electron reduction (yielding CO on pristine In_2_O_3_) to a complex eight-electron reduction that selectively produces CH_4_.^133^ Beyond compositional tuning, controlling morphology and surface structure provides another dimension for optimizing the catalytic performance of In_2_O_3_.

Overall, the enhancement of vacancy-engineered In_2_O_3_ catalysts require a multifaceted approach that transcends simple defect induction. By integrating morphology control, heterojunction fabrication, and the strategic application of cocatalysts, researchers can systematically overcome the limitations inherent in single-component systems. Specifically, morphology control maximizes the exposure of defect-rich active sites; heterojunctions facilitate efficient charge separation through built-in electric fields; and cocatalysts enable essential reactions like hydrogen dissociation and electron pumping. These synergistic strategies do not merely increase vacancy quantity, but rather refine the overall catalytic architecture optimizing reactant adsorption, electron transfer, and structural stability against deactivation. Having established these advanced enhancement strategies, we can now synthesize the broader implications of these developments for the future of CO_2_ reduction.

To contextualize the impact of these design principles, Table 9 summarizes the reported photocatalytic activities of various vacancy-engineered In_2_O_3_ catalysts and representative benchmarks. This comparison reveals a clear performance gap between pristine materials and those subjected to multiscale engineering, underscoring that the integration of morphology control, heterojunctions, and cocatalysts is essential for driving high catalytic productivity.

**Table 9:** Comparison of some In_2_O_3_-based photocatalyst

Category	Material (photocatalytic media)	Key yield	OVs evidence	Stability	References
Baseline	Pristine In_2_O_3_ (water)	CO: 0.52 µmol g^−1^ h^−1^	XPS	4 h	93
			EPR		
			*g* = 2.003		
	Pristine In_2_O_3_ (gas phase)	CO: 11.1 µmol g^−1^ h^−1^	XPS	6 h	134
			EPR		
			*g* = 2.001		
	Pristine In_2_O_3_ (gas phase)	CO: 53.4 µmol g^−1^ h^−1^	XPS	4 h	135
		CH_4_: 11.1 µmol g^−1^ h^−1^	EPR		
			No prominent signal		
	Pristine In_2_O_3_ (gas phase)	CO: <40 µmol g^−1^ h^−1^	XPS	16 h	114
		CH_4_: <20 µmol g^−1^ h^−1^	EPR		
	Pristine In_2_O_3_ (TEOA)	CO: 21.7 µmol g^−1^ h^−1^	EPR	5 h	136 and 137
			XPS		
	Pristine In_2_O_3_ (TEOA)	CO: 12.1 µmol g^−1^ h^−1^	XPS	4 h	130
Morphology control	OVs-In_2_O_3_ nanorods (TEOA)	CO: 63.3 µmol g^−1^ h^−1^	EPR	25 h	137
		CH_4_: 0.8 µmol g^−1^ h^−1^	2.004		
		H_2_: 11.8 µmol g^−1^ h^−1^	XPS		
			531.3 eV		
	H_2_–In_2_O_3_ (OV-rich) (tribo plasma)	CO: 200 µmol g^−1^ h^−1^	EPR	5 cycles	138
			*g* = 2.002		
			XPS		
			529.5 eV		
	In_2_O_3−*x*_ (OH)_*γ*_ (rod-like) (gas phase)	CH_3_OH: 60 µmol g^−1^ h^−1^	XPS	20 h	139
Hetero-junctions	In_2_O_3_@NiIn_2_S_4_	CO: 73.8 µmol g^−1^ h^−1^	EPR: 2.001	16 h	117
	Hollow spheres (gas phase)	CH_4_: 18.5 µmol g^−1^ h^−1^	XPS		
	In_2_O_3_/Bi/BiOCl-4 (water)	CO: 34.53 µmol g^−1^ h^−1^	Raman: new peak at 72 cm^−1^	12 h	140
		CH_4_: 0.89 µmol g^−1^ h^−1^	XPS		
		Selectivity: 97.03%			
	CBB@VO-In_2_O_3_	CO: 130.96 µmol g^−1^ h^−1^	EPR: 2.000	10 cycles	141
	S-scheme (water)		XPS		
	30%OVs-In_2_O_3_/CdSe-DETA (gas phase)	CO: 70.08 µmol g^−1^ h^−1^	EPR	16 h	114
		CH_4_: 27.92 µmol g^−1^ h^−1^			
Cocatalyst	0.2%Au/N–In_2_O_3_/2%Co_3_O_4_ (gas phase)	CO: 96.1 µmol g^−1^ h^−1^	EPR: *g* = 2.001	16 h	135
		CH_4_: 19.9 µmol g^−1^ h^−1^	XPS		
	5 wt% Fe–In_2_O_3_ (water)	CO: 4800 µmol g^−1^ h^−1^ (13.7× increase over In_2_O_3_)	EPR: *g* = 2.002	5 h	40
			XPS: 530.93 eV		
	KIO-3 (K–In_2_O_3_)(OVs) (water)	CO: 3.97 µmol g^−1^ h^−1^	XPS	12 h	93
			EPR *g* = 2.003		
	1.1% Bi–In_2_O_3_ (gas phase)	CO: 125.0 µmol g^−1^ h^−1^	XPS	6 h	134
			EPR *g* = 2.001		
	0.6 wt% Pd–In_2_O_3_ (water)	CO: 45.22 µmol g^−1^ h^−1^	EPR: 2.005	10 h	41
		CH_3_OH: 18.09 µmol g^−1^ h^−1^	PL peaks (450–525 nm)		
		CH_4_: 36.20 µmol g^−1^ h^−1^			
	3.74% N–In_2_O_3_ (TEOA)	CH_3_OH: 394 µmol g^−1^ h^−1^	XPS	30 h	14
	10%Ni–5%Cu/In_2_O_3_	CO: 528.8 µmol g^−1^ h^−1^	XPS	4 cycles	130
		CH_4_: 34.2 µmol g^−1^ h^−1^			
Benchmark material	MnOx/VO-InVO_4_ (water)	CO: 134.6 µmol g^−1^ h^−1^ (selectivity: 92.6%)	EPR	15 h	142
	OVs-rich Pt–TiO_2_ (water)	CO: 54.2 µmol g^−1^ h^−1^	EPR	10 h	143,144
		CH_4_: 66.4 µmol g^−1^ h^−1^	XPS		
			PL: 470 nm		
	OVs-Co_3_O_4_ (TEOA)	CO: 51.7 µmol g^−1^ h^−1^	XPS	12 h	136,145
			EPR		
	40-SnTa_2_O_6−*x*_ nanosheets (gas phase)	CO: 21.1 µmol g^−1^ h^−1^ (visible light)	XPS	5 cycles	146

As illustrated in Table 9, the collective effort in vacancy-engineered catalysis has resulted in substantial progress in boosting C_1_ product yields such as CO, CH_4_, and CH_3_OH through advanced morphological and electronic control. By systematically mapping out these achievements, this newly expanded dataset directly pairs catalytic production rates with verified OV characterization evidence (*e.g.*, *g*-factor assignments *via* EPR and binding energy shifts *via* XPS) alongside their long-term operational stability measured in hours or cycles. However, a persistent bottleneck remains: despite these optimized architectures, the selective formation of C_2+_ products such as ethanol or ethylene remains largely elusive. The tabulated data confirms that while current structural engineering strategies are highly effective at optimizing charge transfer for C_1_ reduction pathways, they are not yet capable of facilitating the complex C–C coupling steps required for multi-carbon generation.

### 2.7. Comparative synthesis of engineering strategies

While the individual structural and electronic impacts of thermal treatment, doping, morphology control, and heterojunction construction have been detailed independently throughout this manuscript, a unified cross-comparison is essential to understand their distinct catalytic behaviors and inherent trade-offs. To provide a holistic overview, Table 10 systematically synthesizes how each core modification strategy simultaneously dictates vacancy density, spatial distribution, band alignments, and long-term operational performance during photocatalytic CO_2_ reduction.

**Table 10:** Comparative evaluation of core modification strategies for vacancy-engineered In_2_O_3_ catalysts

Strategy	Vacancy density	Vacancy location	Band structure	CO_2_ adsorption & activation	Product selectivity	Operational stability
Thermal treatment	Increases with temperature	Bulk-localized at moderate temp	Narrows bandgap	High initial adsorption on OVs site	Poor control	Low
		Surface migration at excessive temp			Favor CO product	Vulnerable to re-oxidation and sintering
Doping	Precise regulation	Surface OVs	Depend on the dopant	Enhanced *via* local electronic polarization	Suppress HER	High
			Introduces donor/acceptor states			Dopants chemically anchor and lock OVs
Morphology control	Facet-dependent	Outermost surface, grain boundaries, and edges	Facet-dependent band edge shifts	Maximized *via* high specific surface area	Dictated by facet coordination and geometry	High in hierarchical or porous frameworks
	Higher in nanostructures *vs.* bulk		Quantum confinement effects			
Heterojunction construction	Highly tunable *via* lattice mismatch strain	Concentrated at boundary interfaces and depletion zones	Establishes built-in electric field	Interfacial charge redistribution enhances activation	Excellent	Robust
			Interfacial electron trapping		Channels continuous electrons for multi-e^−^ steps	Protects core lattice from over-reduction

As synthesized in Table 10, each engineering protocol offers unique advantages accompanied by distinct chemical trade-offs. Thermal treatment and morphology control represent highly direct pathways for generating highly exposed, surface-active vacancies that maximize immediate CO_2_ molecule capture. However, their primary limitations reside in thermodynamic instability and poor target selectivity, which frequently restrains the reaction to simple 2-electron products like CO. Conversely, doping and heterojunction construction serve as sophisticated electronic levers. While they demand more complex synthesis parameters, they successfully anchor active sites to prevent ambient deactivation while simultaneously adjusting the band alignments. This continuous charge redistribution effectively channels photogenerated electrons to drive demanding, multi-electron pathways. Recognizing these multi-parameter dynamics clarifies how the field has successfully optimized single-carbon (C_1_) yields, while highlighting why mastering complex carbon–carbon (C–C) coupling for C_2+_ products remains the next critical bottleneck.

## 3. Conclusion & outlook

In summary, OVs have emerged as the central design parameter governing the photocatalytic performance of In_2_O_3_ for CO_2_ reduction. Beyond acting as simple structural defects, OVs simultaneously regulate the electronic structure, Fermi level position, charge separation, light absorption, CO_2_ adsorption, and reaction kinetics. Throughout this review, we have shown that the catalytic performance of In_2_O_3_ is determined not only by the concentration of oxygen vacancies but also by their spatial distribution (surface *versus* bulk), local coordination environment, clustering behavior, and interaction with other structural features such as heterojunctions, dopants, strain, and SFLPs. Consequently, the current research trend is progressively shifting from maximizing vacancy density toward the rational engineering of well-defined vacancy architectures capable of maintaining high activity while avoiding excessive recombination and catalyst over-reduction.

Despite the remarkable progress achieved during the past decade, several fundamental challenges still hinder the practical implementation of vacancy-engineered In_2_O_3_ photocatalysts. First, the dynamic nature of oxygen vacancies under reaction conditions remains poorly understood. Most characterization studies rely on *ex situ* techniques that capture only the catalyst before or after reaction, whereas oxygen vacancies are continuously generated, annihilated, and redistributed under illumination and reactive atmospheres. Future research should therefore prioritize *operando* and time-resolved characterization techniques including *operando* XPS, DRIFTS, Raman spectroscopy, EPR, X-ray absorption spectroscopy, and environmental TEM to directly monitor vacancy evolution, intermediate formation, and surface reconstruction during photocatalytic CO_2_ reduction. Combining these experimental techniques with DFT calculations and machine-learning-assisted analysis will provide unprecedented insight into the dynamic structure–activity relationships governing catalytic performance.

A second challenge concerns the lack of universally accepted quantitative descriptors for oxygen vacancies. As highlighted throughout this review, different studies employ different methods to estimate vacancy concentration from XPS, Raman, EPR, or PL measurements, making direct comparison between reports extremely difficult. Future studies should move beyond reporting qualitative increases in vacancy concentration and instead establish standardized defect descriptors, including vacancy formation energy, vacancy density per surface area, surface-to-bulk vacancy ratio, vacancy clustering degree, and defect lifetime under operating conditions. Such quantitative descriptors would greatly facilitate the correlation between defect chemistry and photocatalytic performance while improving reproducibility across different laboratories.

Long-term catalyst stability also remains one of the most critical obstacles toward practical deployment. While oxygen vacancies are indispensable active sites, they are intrinsically dynamic and susceptible to migration, agglomeration, reoxidation, or excessive reduction into metallic indium under prolonged operation. Future catalyst design should therefore emphasize stabilization strategies that preserve highly active surface vacancies while preventing structural degradation. Rational approaches including dopant anchoring, interfacial confinement, heterojunction engineering, strain engineering, and controlled vacancy clustering appear particularly promising for maintaining catalytic activity over extended operating times without sacrificing defect functionality.

Another major research frontier concerns product selectivity. Current vacancy-engineered In_2_O_3_ photocatalysts predominantly favor C_1_ products such as CO, CH_4_, and CH_3_OH because oxygen vacancies efficiently facilitate CO_2_ adsorption and the early hydrogenation steps. However, selective production of higher-value C_2+_ products remains extremely limited owing to the high kinetic barrier associated with C–C coupling. Future research should therefore focus on multifunctional catalytic architectures capable of simultaneously stabilizing key reaction intermediates, promoting multi-electron transfer, and facilitating C–C bond formation. Synergistic integration of oxygen vacancies with S-scheme or Z-scheme heterojunctions, plasmonic cocatalysts, single-atom active sites, SFLPs, and tandem catalytic systems may provide effective pathways toward selective production of multicarbon fuels and chemicals.

Finally, translating laboratory-scale discoveries into practical solar fuel technologies will require addressing several scale-up challenges. Future research should extend beyond powder catalyst synthesis toward scalable fabrication methods, continuous-flow photoreactors, efficient photon utilization, catalyst immobilization, large-area photoelectrode integration, and long-term outdoor operation under realistic solar irradiation. Economic considerations, catalyst durability, raw material availability, and life-cycle assessment should also become integral components of future research to evaluate the true potential of vacancy-engineered In_2_O_3_ for industrial CO_2_ conversion.

Overall, the future of In_2_O_3_ photocatalysis lies not in simply creating more oxygen vacancies but in precisely controlling their structure, location, dynamics, and interaction with surrounding catalytic environments. The convergence of *operando* characterization, quantitative defect engineering, computational materials design, standardized evaluation protocols, and scalable catalyst fabrication is expected to transform oxygen-vacancy engineering from an empirical optimization strategy into a predictive design principle for next-generation photocatalysts capable of efficient, selective, stable, and economically viable solar-driven CO_2_ conversion.

## Conflicts of interest

The authors declare that they have no competing interests.

## Abbreviations

BRANNs: Bayesian regularized artificial neural network
CBM: Conduction band minimum
CO_2_RR: CO_2_ reduction reaction
DFT: Density functional theory
DOS: Density of state
DSHDs: Double-shell hollow dodecahedrons
DRIFTS: Diffuse reflectance infrared fourier transform spectroscopy
*E*_a_
: Activation energy
EELS: Electron energy loss spectroscopy
*E*_f_
: Fermi energy
EIS: Electrochemical impedance spectroscopy
EPR: Electron paramagnetic resonance
FE: Faradaic efficiency
GO: Graphene oxide
HAADF-STEM: High angle annular dark filed-scanning transmission electrons microscopy
HER: Hydrogen evolution reaction
HRTEM: High-resolution transmission electron microscopy
H2-TPM: Hydrogen temperature-programmed reduction
LMP: Liquid metal printing
OVs: Oxygen vacancies
ML: Machine learning
MO_*x*_: Semiconductor metal oxides
PL: Photoluminescence
PRO: Partially reducible oxide
RWGS: Reverse water gas shift
SFLPs: Surface frustrated lewis pairs
STO: Strontium titanate
Sv: Sulfur vacancy
TCO: Transparent conducting oxide
VBM: Valence band maximum
XPS: X-ray photoelectron spectroscopy

## Data Availability

No primary research results, software or code have been included and no new data were generated or analyzed as part of this review.
